# Human hantavirus infection elicits pronounced redistribution of mononuclear phagocytes in peripheral blood and airways

**DOI:** 10.1371/journal.ppat.1006462

**Published:** 2017-06-22

**Authors:** Saskia Scholz, Faezzah Baharom, Gregory Rankin, Kimia T. Maleki, Shawon Gupta, Sindhu Vangeti, Jamshid Pourazar, Andrea Discacciati, Jonas Höijer, Matteo Bottai, Niklas K. Björkström, Johan Rasmuson, Magnus Evander, Anders Blomberg, Hans-Gustaf Ljunggren, Jonas Klingström, Clas Ahlm, Anna Smed-Sörensen

**Affiliations:** 1 Immunology and Allergy Unit, Department of Medicine Solna, Karolinska Institutet, Karolinska University Hospital, Stockholm, Sweden; 2 Department of Public Health and Clinical Medicine, Division of Medicine, Umeå University, Umeå, Sweden; 3 Center for Infectious Medicine, Department of Medicine Huddinge, Karolinska Institutet, Karolinska University Hospital, Stockholm, Sweden; 4 Unit of Biostatistics, Institute of Environmental Medicine, Karolinska Institutet, Stockholm, Sweden; 5 Department of Clinical Microbiology, Infectious Diseases, Umeå University, Umeå, Sweden; 6 Department of Clinical Microbiology, Virology, Umeå University, Umeå, Sweden; Division of Clinical Research, UNITED STATES

## Abstract

Hantaviruses infect humans via inhalation of virus-contaminated rodent excreta. Infection can cause severe disease with up to 40% mortality depending on the viral strain. The virus primarily targets the vascular endothelium without direct cytopathic effects. Instead, exaggerated immune responses may inadvertently contribute to disease development. Mononuclear phagocytes (MNPs), including monocytes and dendritic cells (DCs), orchestrate the adaptive immune responses. Since hantaviruses are transmitted via inhalation, studying immunological events in the airways is of importance to understand the processes leading to immunopathogenesis. Here, we studied 17 patients infected with Puumala virus that causes a mild form of hemorrhagic fever with renal syndrome (HFRS). Bronchial biopsies as well as longitudinal blood draws were obtained from the patients. During the acute stage of disease, a significant influx of MNPs expressing HLA-DR, CD11c or CD123 was detected in the patients’ bronchial tissue. In parallel, absolute numbers of MNPs were dramatically reduced in peripheral blood, coinciding with viremia. Expression of CCR7 on the remaining MNPs in blood suggested migration to peripheral and/or lymphoid tissues. Numbers of MNPs in blood subsequently normalized during the convalescent phase of the disease when viral RNA was no longer detectable in plasma. Finally, we exposed blood MNPs *in vitro* to Puumala virus, and demonstrated an induction of CCR7 expression on MNPs. In conclusion, the present study shows a marked redistribution of blood MNPs to the airways during acute hantavirus disease, a process that may underlie the local immune activation and contribute to immunopathogenesis in hantavirus-infected patients.

## Author summary

Inhalation of hantavirus-infected rodent droppings can cause a wide range of disease ranging from mild symptoms to deaths in humans. Central to hantavirus disease is vascular leakage that can manifest in different organs, including the lungs. Although the virus can infect endothelial cells lining the blood vessels, it does not cause cell death. Instead, activation of the immune system in response to viral infection has been implicated in causing vascular leakage. In this study, we investigated how monocytes and dendritic cells (DCs) are involved in hantavirus disease, given their capacity to activate other immune cells. We obtained unique clinical material from 17 Puumala virus-infected patients including mucosal biopsies from the airways as well as multiple blood draws over the course of disease. In the airways of these patients, we observed an infiltration of monocytes and DCs. In parallel, there was a dramatic depletion in peripheral blood—more than ten-fold—of monocytes and DCs that was sustained throughout the first two weeks of disease. Taken together, this study provides novel insights into immune mediated processes underlying human hantavirus pathogenesis.

## Introduction

Hantaviruses pathogenic to humans are rodent borne, but do not cause disease in their natural hosts. However, transmission to humans via inhalation of aerosolized virus-contaminated rodent excreta may lead to severe disease and death, thus representing a severe threat to public health [1, 2]. Hantaviruses in Europe and Asia primarily cause hemorrhagic fever with renal syndrome (HFRS) whereas hantaviruses in the Americas cause hantavirus pulmonary syndrome (HPS), with case fatality rates of 0.1–10% and 40% respectively [3]. Puumala virus (PUUV), the endemic strain in Sweden, has an incubation time of 2–3 weeks and can cause a mild form of HFRS, also referred to as *nephropathia epidemica* [2, 4, 5]. In humans, hantaviruses infect the vascular endothelium without causing cytopathic effects [6]. Yet, increased vascular permeability is a hallmark of hantavirus diseases. It has been suggested that an immune-mediated dysregulation of endothelial permeability might contribute to disease pathogenesis [1, 3, 7–9]. Hantavirus immunopathogenesis is most likely a complex multifactorial process involving both innate [10–12] and adaptive immune cells [13–15]. Cytotoxic T lymphocytes (CTLs) and natural killer (NK) cells as well as pro-inflammatory cytokines such as tumor necrosis factor (TNF) produced by these lymphocytes have been implicated in causing capillary leakage [16]. Supporting this notion, stronger CTL responses have been associated with a more severe disease outcome and even death [14, 17–20].

Monocytes and dendritic cells (DCs), together termed mononuclear phagocytes (MNPs), are able to present viral antigens to T cells, thus initiating and regulating virus-specific immune responses [21, 22]. In human blood, monocytes can be further subdivided into classical, intermediate and non-classical monocytes based on varying expressions of CD14 and CD16 [23]. During both bacterial and viral infections, intermediate and non-classical monocytes in blood of patients have been reported to increase in numbers [24–27]. Kwissa *et al*. further illustrated in acute dengue virus infection that the expansion of intermediate monocytes correlated with formation of plasmablasts, important for development of humoral responses [27]. Indeed, a robust production of hantavirus-specific plasmablasts in circulation, as well as IgG and IgM antibodies in serum may be necessary for patient recovery and even survival [28–30].

DCs, which are superior to monocytes in priming naïve T cell responses, consist of plasmacytoid DCs (PDCs) and myeloid DCs (MDCs) that can be further separated into CD1c^+^ MDCs and CD141^+^ MDCs [31, 32]. During viral infections, DCs are rapidly mobilized to peripheral tissues where they replenish the tissue-resident DCs that first encounter the pathogens and either die due to infection or migrate to draining lymph nodes [33–35]. In humans, both monocyte-derived cells as well as DCs have been observed in respiratory compartments at steady state with the capacity to detect and respond to invading pathogens [31, 36–39]. Since hantaviruses are transmitted predominantly via inhalation, studying immunological events in the airways where viral replication is first initiated is of importance to understand the processes leading to immunopathogenesis. Furthermore, pulmonary dysfunction has been reported in HFRS patients [40–42]. Expansion of cytotoxic CD8^+^ T cells in the airways of hantavirus-infected patients has been described as contributing to disease severity [13, 14]. This suggests that DCs may be involved in promoting the recruitment and local activation of T cells. However, little is known on the contributions of MNPs in human hantavirus-infected patients *in vivo*, especially at the site of entry.

In order to investigate the involvement of monocytes and DCs in local immune events in the airways, we obtained endobronchial biopsies from 17 PUUV-infected HFRS patients during the acute phase of disease and compared them to samples from uninfected controls (UC). We illustrated a significant infiltration of CD8^+^ T cells and MNPs into the bronchial tissue during acute HFRS compared to UC. As hantaviruses establish a systemic infection, we characterized MNPs in longitudinal peripheral blood samples from the same patients. Concurrent with the increase of MNPs in the airways, we observed a dramatic depletion of circulating monocytes and DCs during the acute phase of disease. By investigating the distribution of monocytes and DCs in different anatomical compartments during an acute viral infection in humans, we gained insights into the potential roles of specific cell subsets based on their migratory patterns and tissue-specific locations during the course of disease.

## Results

### Hantavirus patients experienced respiratory problems

**Fig 1:**
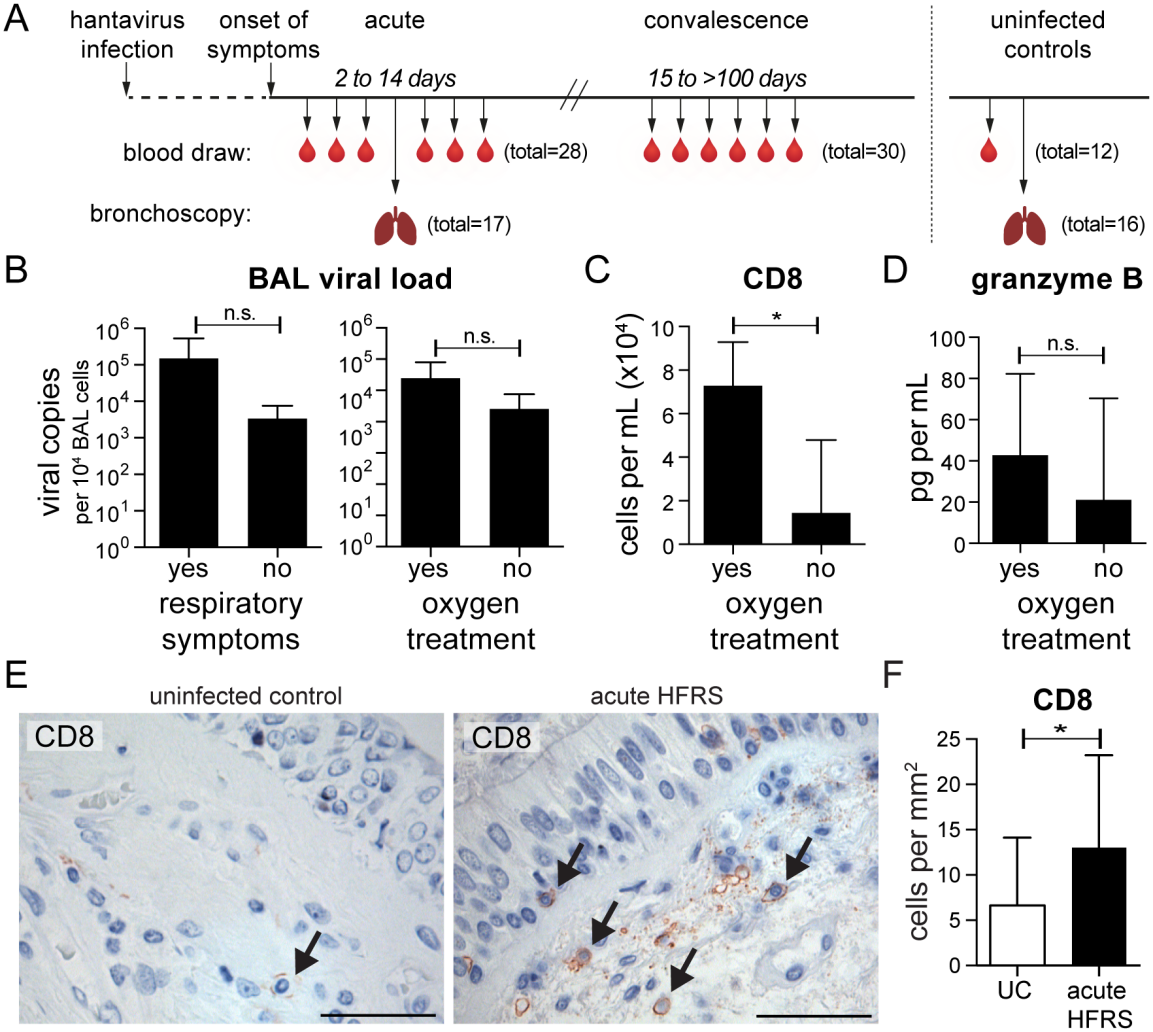
Respiratory involvement in hantavirus-infected patients. **(A)** Hantavirus-infected patients diagnosed with hemorrhagic fever with renal syndrome (HFRS patients) underwent bronchoscopy (median day 9, n = 17) for collection of endobronchial biopsies and bronchoalveolar lavage (BAL). Multiple blood draws were taken from 12 patients during acute (median day 6, total of all patients = 28) and convalescent (median day 89, total of all patients = 30) phases. Uninfected controls (UC) were sampled for blood (n = 12) and endobronchial biopsies (n = 16). **(B)** Detectable viral load in BAL cells of 15 patients were further classified according to whether they experienced respiratory symptoms (left panel) or needed oxygen treatment (right panel). Bar graphs show mean±SD. **(C)** Mean±SD absolute numbers of CD8^+^ T cells were evaluated by flow cytometry in BAL of patients with or without oxygen treatment. **(D)** Granzyme B was measured in BAL fluid of patients with (n = 5) or without (n = 12) oxygen treatment and presented as mean±SD. **(E)** Representative images of CD8 staining in endobronchial biopsies from UC (n = 16) and HFRS patient (n = 17). Specific staining appears in red, and cell nuclei are counterstained with hematoxylin in blue. Arrows indicate positive staining. Visualization was performed using immunohistochemistry. Scale bar, 50 μm. **(F)** Bar graph summarizes mean±SD comparing HFRS patients (n = 17) during the acute phase of infection with UC (n = 16). The number of CD8^+^ cells is expressed as cells/mm^2^. Statistical differences were assessed using two-tailed Mann–Whitney U-test: * *p*<0.05, n.s. not significant.

Although HFRS mainly manifests in the kidneys, respiratory involvement has been increasingly documented in these patients, including pulmonary edema that may lead to respiratory failure [14, 28, 40]. Of the 17 PUUV-infected HFRS patients included in this study, 10 experienced respiratory symptoms such as dry cough and dyspnea, and 5 needed oxygen treatment (S1 Table). Given that the airways are the initial site of infection after inhalation of hantavirus-contaminated rodent excreta, little is known regarding the early immune response taking place locally. In the current study, bronchoscopy was performed on PUUV-infected HFRS patients in order to sample their airways during the acute phase of disease. As soon as platelet counts had stabilized and the patients were able to withstand the procedure, endobronchial biopsies and bronchoalveolar lavage (BAL) were collected from each patient (median 9 days after onset of symptoms). In addition, longitudinal peripheral blood samples were collected from these patients during both the acute phase (2–14 days after onset of symptoms) and convalescent phase (>15 days after onset of symptoms) of HFRS. Results from blood and lung samples collected from HFRS patients were compared throughout the study with samples from UC, who similarly underwent bronchoscopies and blood draws (Fig 1A). In 15 patients, viral load was detected in BAL cells, suggesting local viral replication. However, viral load alone could neither explain the respiratory symptoms experienced by 10 patients, nor the need for oxygen treatment by 5 patients (Fig 1B). As CTLs have been implicated in contributing to hantavirus pathogenesis [13, 14, 16, 43], we investigated the absolute numbers of CD8^+^ T cells in the airways of patients. As previously described within the same study cohort [14], more CD8^+^ T cells were detected in BAL of patients that required oxygen treatment when compared to those who did not need oxygen treatment (*p*<0.05) (Fig 1C). Granzyme B, a cytotoxic protease released by CTLs and NK cells that can induce apoptosis, was also detected in BAL fluid of patients, with a trend towards higher amounts of granzyme B in patients requiring oxygen treatment (n = 5), although the difference was not significant (*p* = 0.08) (Fig 1D), possibly due to the relatively large variation between individuals. In order to assess whether CD8^+^ T cells were also present in bronchial tissue of patients, we performed immunohistochemistry on sections of endobronchial biopsies (Fig 1E). Indeed, significantly more CD8^+^ T cells were detected in biopsies taken from patients with acute HFRS than in biopsies from UC (Fig 1F). This prompted us to examine aspects of local T cell activation by investigating monocytes and DCs in the airways during hantavirus disease.

### Infiltration of MNPs in the airways during acute HFRS

**Fig 2:**
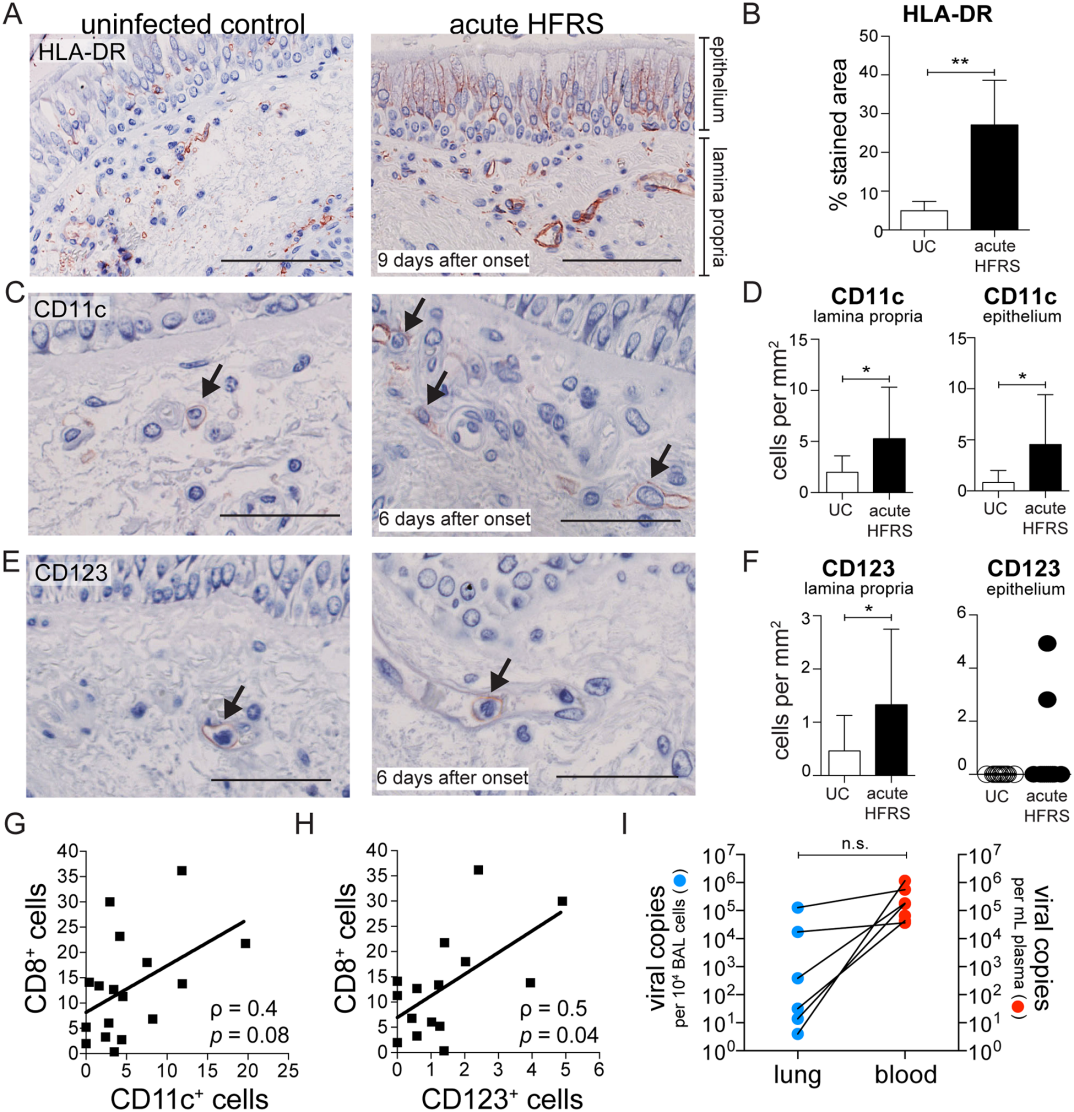
Infiltration of MNPs into the airways during acute hantavirus infection. Endobronchial biopsies were taken from patients with acute HFRS and age- and sex-matched UC. (**A**) Representative images of endobronchial biopsies revealing high numbers of HLA-DR^+^ cells in HFRS patients (n = 5), as compared to UC (n = 5) are shown. Specific staining appears in red, and cell nuclei are counterstained with hematoxylin in blue. Arrows indicate positive staining. Visualization was performed using immunohistochemistry. Scale bar, 100 μm. (**B**) Bar graph summarizes mean±SD percentage of HLA-DR stained areas of all subjects. Representative images of (**C**) CD11c and (**E**) CD123 staining in lamina propria and epithelium in endobronchial biopsies from HFRS patients and UC. Scale bar, 50 μm. (**D** and **F**) Bar graphs summarize mean±SD comparing HFRS patients (n = 17) during the acute phase of infection with UC (n = 16). The numbers of CD11c^+^ or CD123^+^ cells are expressed as cells/mm^2^ of epithelium and lamina propria, respectively. Statistical differences were assessed using two-tailed Mann–Whitney U-test: * *p*<0.05 ** *p*<0.01. Graphs show the positive correlation (ρ) of CD8^+^ cells to (**G**) CD11c^+^ cells and (**H**) CD123^+^ cells in endobronchial biopsies of HFRS patients as assessed by Spearman’s correlation test (n = 17). (**I**) Detectable viral load in BAL cells (per 10^4^ cells) and plasma (per mL) of HFRS patients measured by viral RNA quantification on the day of bronchoscopy. Lines indicate paired measurements in BAL cells and plasma of individual patients (n = 8).

By immunohistochemistry, we investigated the distribution and frequency of cells expressing surface markers for monocytes and DCs on sections of endobronchial biopsies. We found significantly more HLA-DR^+^ cells (*p*<0.01) in biopsies taken from patients with acute HFRS than in biopsies from UC (Fig 2A and 2B). The increased HLA-DR staining, especially in the lamina propria, suggested an infiltration of MNPs. Additionally, the pulmonary epithelium also displayed increased HLA-DR staining in tissues from acute HFRS patients, possibly from local inflammation leading to upregulation of HLA-DR on epithelial cells [44] in addition to infiltration of immune cells to the site of infection. We also observed a significant increase in the number of cells expressing the myeloid cell marker CD11c during acute HFRS compared to UC (Fig 2C). Detailed analysis of bronchial tissue showed significantly increased numbers of CD11c^+^ cells in the lamina propria (*p*<0.05) and epithelium (*p*<0.05) of hantavirus-infected patients compared to UC (Fig 2D). In addition, the number of cells expressing the PDC marker CD123 was also significantly higher in the lamina propria (*p*<0.05) of these patients (Fig 2E and 2F). To address whether patients with high numbers of CD8^+^ T cells in the bronchial tissue also had high numbers of MNPs, we performed a Spearman correlation test and observed a positive association between CD8^+^ cells and CD11c^+^ cells (*p* = 0.08) (Fig 2G), and a significant correlation between CD8^+^ cells and CD123^+^ cells (*p*<0.05) (Fig 2H). Taken together, we observed an infiltration of MNPs into the airways during acute HFRS coinciding with the presence of CD8^+^ T cells at the site of infection. However, hantavirus infection is typically systemic and not limited to the airways [3]. Indeed, viral RNA copies can be detected in the plasma early during disease onset, but also on the day of bronchoscopy when the bronchial biopsies were obtained (Fig 2I and S1 Fig). Thus, we next explored the possibility that blood DCs and monocytes exposed to virus or virus-induced cytokines may have received signals to migrate into the airways, contributing to the significant influx of MNPs in the bronchi.

### Blood monocytes were reduced in patients with acute HFRS

**Fig 3:**
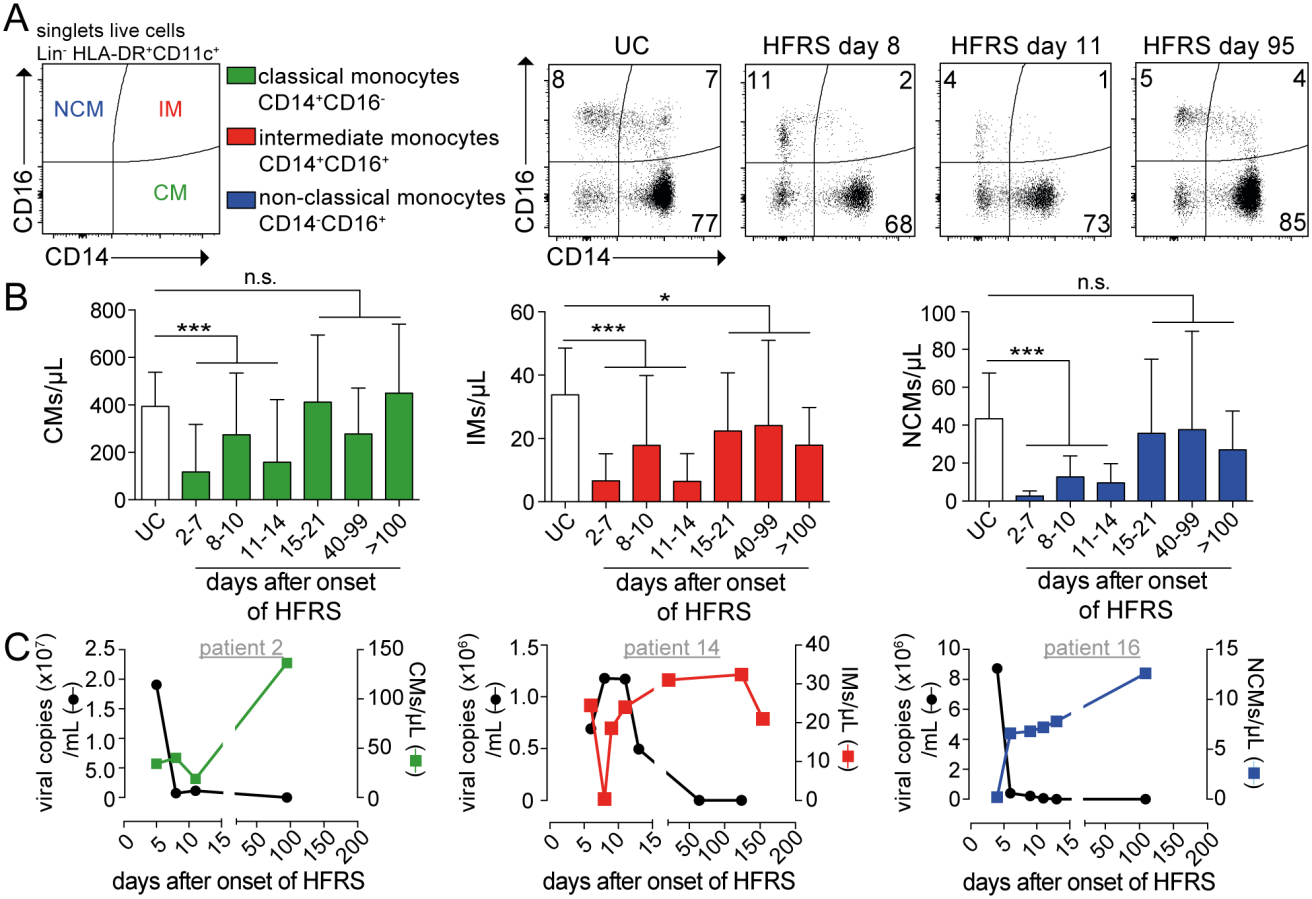
Reduction in absolute numbers of human monocytes in circulation during acute HFRS. **(A)** Gating strategy for the identification of classical monocytes (CM, green), intermediate monocytes (IM, red) and non-classical monocytes (NCM, blue) by flow cytometry after gating out lineage (CD3, CD20, CD56) cells and gating on HLA-DR^+^, CD11c^+^ cells. Representative flow cytometry plots from one representative UC and HFRS patient are shown. **(B)** Mean±SD absolute numbers of monocyte populations were evaluated in longitudinal samples in HFRS patients. **(C)** Graphs show detectable viral load in plasma measured by viral RNA quantification from one representative HFRS patient. Virological data (left axis; black line) and absolute numbers of monocytes (right axis; colored line) plotted over time from one representative HFRS patient. Differences in mean absolute number of monocytes were assessed using Poisson regression: *** *p*<0.001, n.s. not significant.

Since monocytes participate in inflammation [22], especially following viral infection, we hypothesized that the number of monocytes would expand in peripheral blood during the acute phase of HFRS, as has been observed in other acute viral infections [27, 45, 46]. To investigate how hantavirus infection may affect monocytes in circulation, the frequencies of classical (CD14^+^ CD16^-^), intermediate (CD14^+^ CD16^+^) and non-classical (CD14^-^ CD16^+^) monocytes were analyzed using flow cytometry (Fig 3A and S2 Fig). The absolute number of classical monocytes per microliter of blood decreased significantly (*p*<0.001) during acute HFRS compared to samples from UC (Fig 3B and S2 Table), and subsequently normalized during the convalescent phase of the disease (S3 Table). Similarly, the numbers of intermediate monocytes and non-classical monocytes were also significantly reduced (*p*<0.001) during acute HFRS compared to UC (Fig 3B and S2 Table). Interestingly, for all the monocyte subsets observed, the low numbers of cells in circulation coincided with high viral load (viral RNA copies per mL of plasma) as assessed by quantitative reverse transcriptase polymerase chain reaction (qRT-PCR) (Fig 3C and S1 Fig). During convalescence, when virus was no longer detectable in plasma, the number of monocytes returned to comparable values as those in UC, except for intermediate monocytes. In summary, we observed a loss of monocytes in the peripheral blood of patients during the acute phase of HFRS.

### DC subsets were diminished in blood during acute HFRS

**Fig 4:**
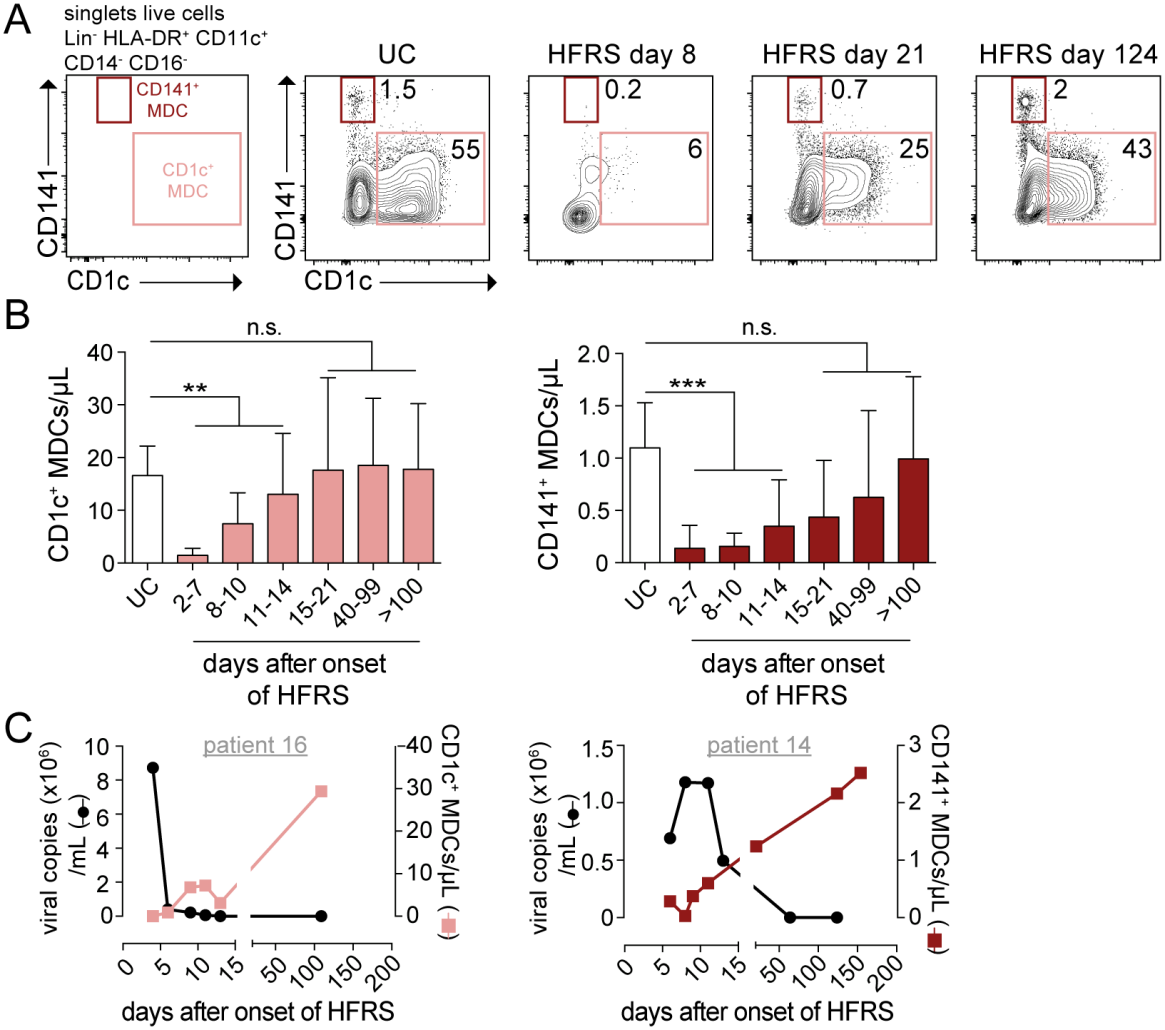
Decreased numbers of CD1c^+^ and CD141^+^ MDCs in blood during acute HFRS. (**A**) Gating strategy for the identification of CD1c^+^ MDCs (coral) and CD141^+^ MDCs (maroon) by flow cytometry after gating out lineage (CD3, CD20, CD56) cells, gating on HLA-DR^+^, CD11c^+^ cells, and gating out monocytes (CD14, CD16). Representative flow cytometry plots from one representative UC and HFRS patient are shown. (**B**) Mean±SD absolute numbers of MDC populations were evaluated in longitudinal samples in HFRS patients in comparison to UC. (**C**) Graphs show detectable viral load in plasma measured by viral RNA quantification from one representative HFRS patient. Virological data (left axis; black line) and absolute numbers of MDCs (right axis; colored line) plotted over time from one representative HFRS patient. Differences in mean absolute number of MDCs were assessed using Poisson regression: ** *p*<0.01, *** *p*<0.001, n.s. not significant.

The loss of monocytes in circulation during acute HFRS led us to also assess whether DCs, key determinants of viral disease outcome due to their capacity to initiate and activate T cell responses [47], would also be affected by hantavirus infection. We first analyzed the two MDC subsets found in human blood: CD1c^+^ MDCs and CD141^+^ MDCs (Fig 4A). We observed a dramatic reduction of both MDC subsets during acute HFRS as compared to UC (Fig 4A). The reduction in absolute numbers was statistically significant for CD1c^+^ MDCs (p<0.01) and CD141^+^ MDCs (p<0.001) (Fig 4B). On average, the number of CD1c^+^ MDCs was as low as 1.5 cells per microliter of blood during the early acute phase as compared to 16 cells per microliter found in UC (S2 Table). The number of CD141^+^ MDCs, already rare under steady state conditions, decreased by 97% during acute HFRS (S3 Table). For both CD1c^+^ and CD141^+^ MDCs, the cell numbers normalized during the convalescent phase of the disease (Fig 4B). Of importance, we excluded the possibility that the reduced number of DCs in acute HFRS blood samples was a consequence of the cells being more fragile to freeze-thawing, by confirming similarly low frequencies of DCs and monocytes in fresh samples from patients with acute HFRS (S3 Fig). As with the monocytes, we compared the kinetics of plasma viral load with the absolute numbers of MDCs in blood and found that high viral load coincided with low numbers of CD1c^+^ and CD141^+^ cells (Fig 4C).

**Fig 5:**
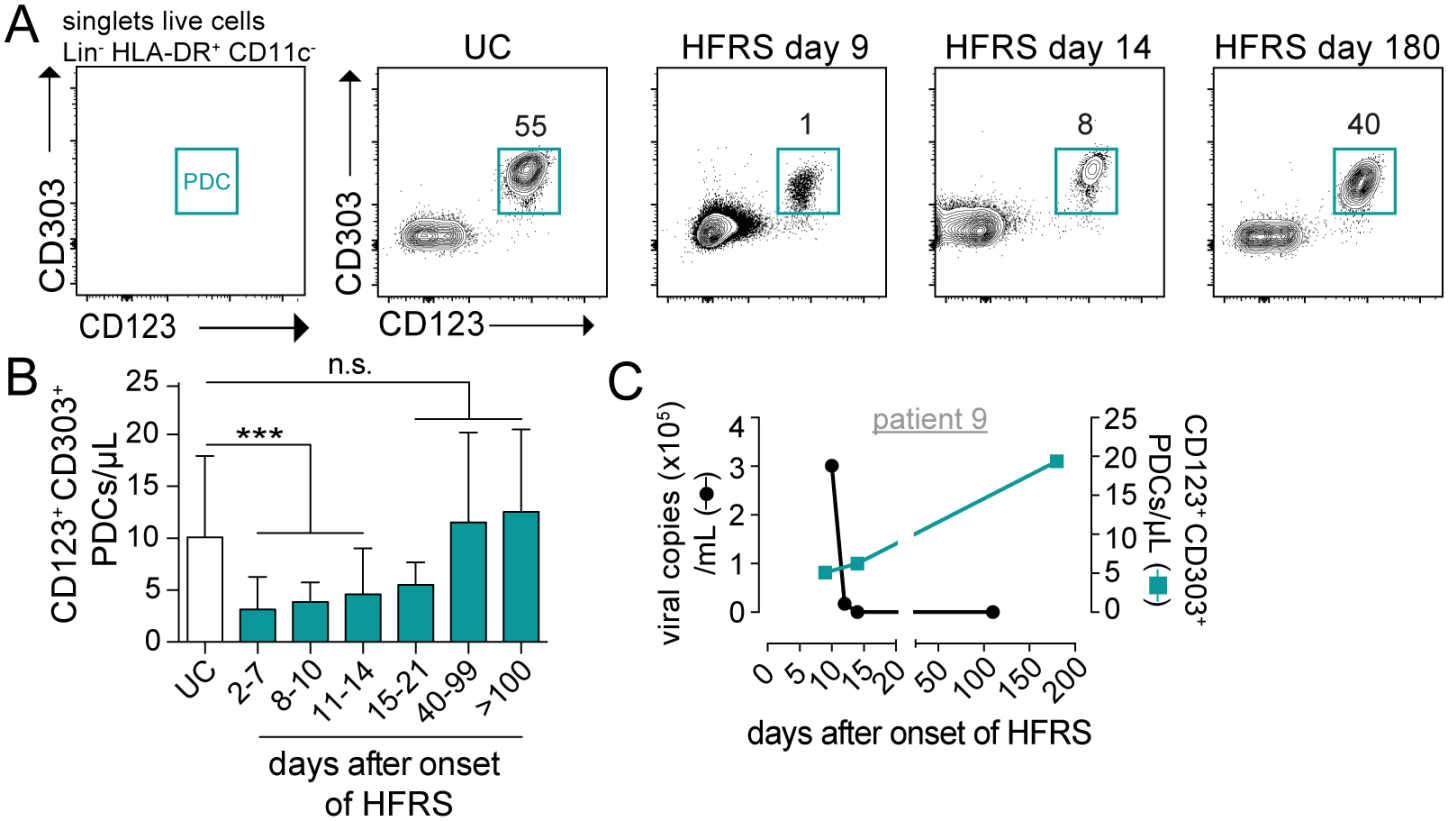
Decreased number of PDCs in blood during acute HFRS. (**A**) Gating strategy for the identification of PDCs (teal) by flow cytometry after gating out lineage (CD3, CD20, CD56) cells, and gating on HLA-DR^+^ CD11c^-^ cells. Representative flow cytometry plots from one representative UC and HFRS patient are shown. (**B**) Mean±SD absolute numbers of PDCs were evaluated in longitudinal samples in HFRS patients in comparison to UC. (**C**) Graphs show detectable viral load in plasma measured by viral RNA quantification from one representative HFRS patient Virological data (left axis; black line) and absolute numbers of PDCs (right axis; colored line) are shown over time from one representative HFRS patient. Differences in mean absolute number of PDCs were assessed using Poisson regression: *** *p*<0.001, n.s. not significant.

We also assessed the numbers of PDCs in blood during acute and convalescent HFRS. PDCs are the major producers of antiviral type I interferon (IFN) in the body and are important in the defense against viral pathogens, despite their low frequency. Yet, levels of IFN-α are not elevated in blood of HFRS patients [48]. Here, we found that the number of blood PDCs, as defined by their CD123 and CD303 expression, was significantly reduced during acute HFRS as compared to UC (Fig 5A). The drop in absolute PDC number (p<0.001) was maintained also during early convalescence at days 15–21 after the onset of HFRS, but eventually returned to normal values (Fig 5B and S2 Table). Again, the loss of blood PDCs during acute HFRS coincided with high viral load (Fig 5C). Together, a massive depletion of both MDCs and PDCs was observed in peripheral blood during acute HFRS.

### Upregulation of CCR7 on blood monocytes and DCs in HFRS patients

**Fig 6:**
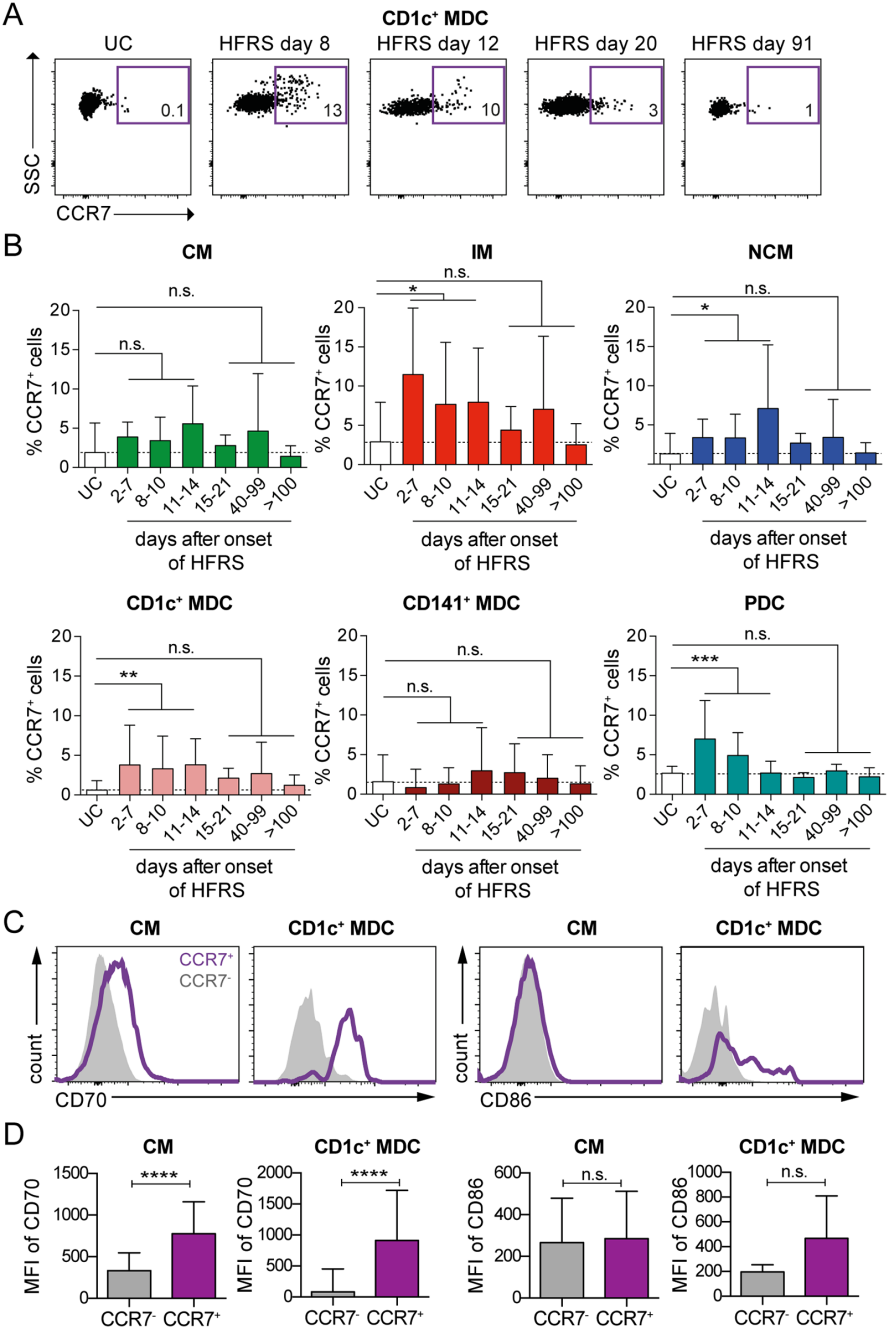
Upregulation of CCR7 on MNPs in blood of HFRS patients during acute infection. (**A**) Representative flow cytometry plots showing CCR7 expression on CD1c^+^ MDCs from one representative UC and one HFRS patient are shown. (**B**) Mean±SD frequencies of CCR7^+^ CMs (green), IM (red), NCM (blue), CD1c^+^ MDCs (coral), CD141^+^ MDCs (maroon) and PDCs (teal) were quantified in longitudinal samples from HFRS patients in comparison to UC. (**C**) Histograms indicate upregulation of CD70 on CM, and upregulation of CD86 and CD70 on CD1c^+^ MDCs of a HFRS patient during the acute phase. (**D**) Bar graphs summarize the MFI±SD of CD70 and CD86 in CCR7^+^ and CCR7^-^ CM and CD1c^+^ MDCs. Differences in proportion of CCR7^+^ cells were assessed using logistic regression; * *p*<0.05, ** *p*<0.01, *** *p*<0.001, n.s. not significant.

As hantaviruses are not known to cause cytopathic effects, the loss of monocytes and DCs in circulation could reflect a redistribution of MNPs from circulation into the airways, as we had observed an infiltration of MNPs in the bronchial tissue (Fig 2). To assess whether trafficking of blood DCs and monocytes to lymph nodes [49, 50] or other tissues [51, 52] could account for the reduced numbers of monocytes and DCs in blood during acute HFRS, we measured the surface expression of the chemokine receptor CCR7 on the few MNPs still in circulation. At steady state, few or no cells expressed CCR7, as exemplified by the UC (Fig 6A). However, during acute HFRS, a subset of the cells remaining in peripheral blood expressed CCR7 on their surfaces that progressively disappeared over time, as exemplified by CD1c^+^ MDCs (Fig 6A). Intermediate monocytes and non-classical monocytes presented with a higher frequency of CCR7^+^ cells in HFRS patients compared to UC (*p*<0.05) during acute HFRS (Fig 6B). CD1c^+^ MDCs (*p*<0.01) also upregulated CCR7 expression pattern throughout the acute phase, while the CD141^+^ MDCs showed no or very modest upregulation of CCR7 (Fig 6B). Although a subset of PDCs upregulated CCR7 during early acute HFRS (*p*<0.001), the frequency of CCR7^+^ PDCs returned back to low levels in the late stage of acute disease (days 11–14), at frequencies similar to those in UC (Fig 6B).

Although the overall maturation profile of monocytes and DCs in circulation as determined by upregulation of co-stimulatory molecules CD70 and CD86 was not pronounced, CCR7^+^ cells had a higher expression of CD70 (classical monocytes and CD1c^+^ MDCs) and CD86 (CD1c^+^ MDCs) than the CCR7^-^ cells, consistent with a more mature phenotype (Fig 6C and 6D). Taken together, the data suggest that although the majority of monocytes and DCs are absent in circulation during acute HFRS, the cells that remain in blood appear to have received signals to upregulate migratory receptors such as CCR7, facilitating migration to lymph nodes or peripheral tissues.

### Blood monocytes and CD1c^+^ MDCs were susceptible to PUUV infection *in vitro* and upregulated CCR7 after viral exposure

**Fig 7:**
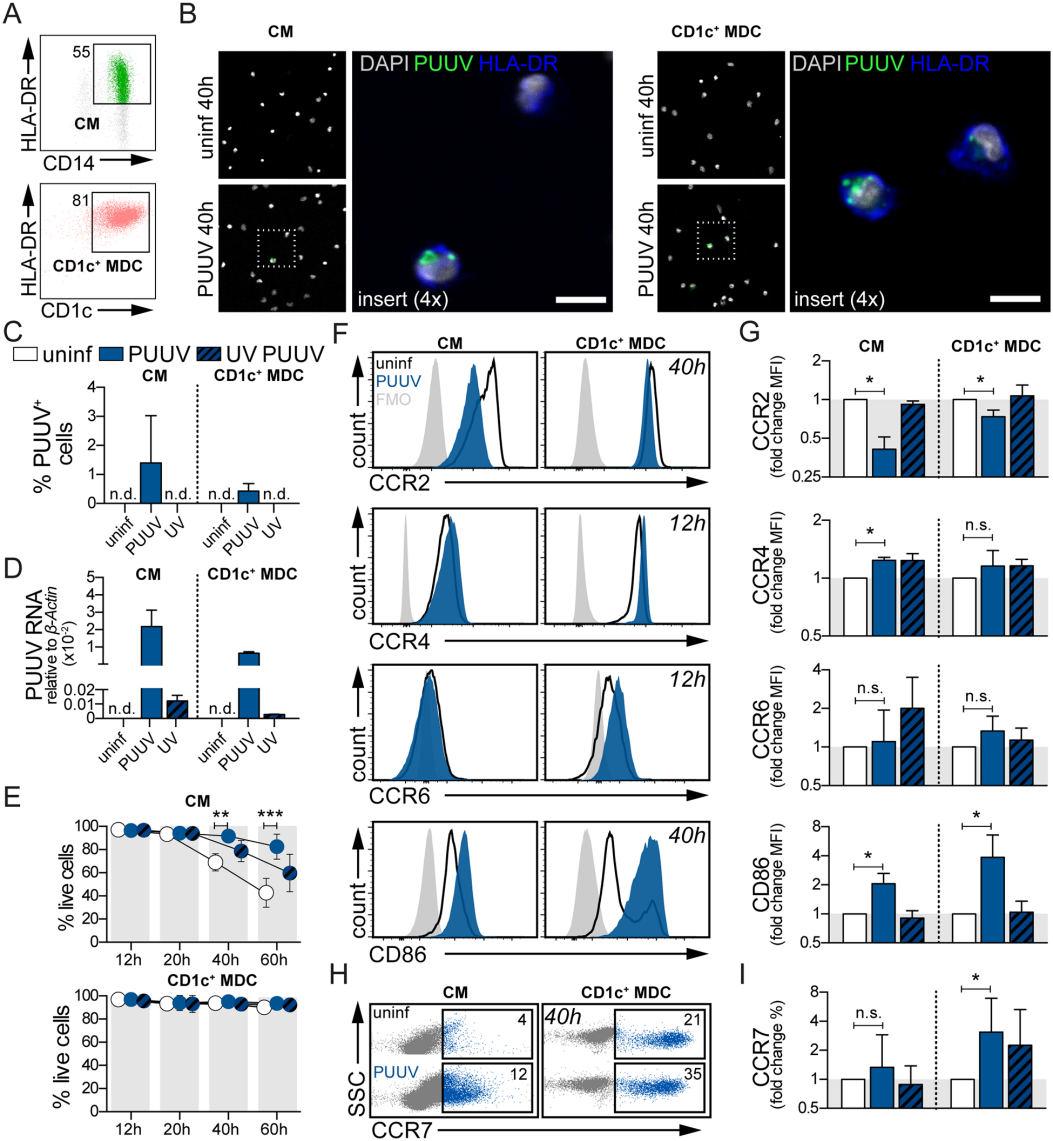
Susceptibility of classical monocytes and CD1c^+^ MDCs to PUUV *in vitro*. (**A**) Human CM (top panel, green) and CD1c^+^ MDCs (bottom panel, coral) were isolated from peripheral blood of healthy volunteers. Flow cytometry dot plots show live, HLA-DR^+^ CD11c^+^ CD14^+^ CD16^-^ monocytes or live, CD11c^+^ CD1c^+^ MDCs. Numbers in gate depict the frequency of cells out of total live cells. One representative donor of six is shown. Cells were left unexposed, exposed to PUUV or UV-inactivated PUUV for 2 h at an MOI of 7.5. Cells were washed and subsequently incubated for 12–60 h. (**B**) Immunofluorescence staining with patient serum on CM (left panel) and CD1c^+^ MDCs (right panel) on uninfected and PUUV-infected cells 40 h after infection indicates detectable viral antigen (green). Cells were counterstained with DAPI (gray) and also stained for HLA-DR (blue). Scale bar, 10 μm. (**C**) Bar graphs summarize the mean±SD of PUUV^+^ CM (left panel, n = 3) or CD1c^+^ MDCs (n = 3). n.d., not detectable. (**D**) Relative expression of PUUV RNA was measured in CM and CD1c^+^ MDCs (n = 2) after 60 h of infection. Bar graphs show 2^-ΔCt^ values relative to the housekeeping gene *β-Actin*. (**E**) Viability of cells was assessed by flow cytometry based on a LIVE/DEAD dye. Graphs show mean±SD viability of CM (top panel, n = 4) or CD1c^+^ MDC (bottom panel, n = 6). (**F**) Histograms indicate changes in expression of CCR2, CCR4, CCR6 and CD86 in cells exposed to PUUV (ocean blue) compared to unexposed (black line) from one representative donor of CM (n = 4) and CD1c^+^ MDCs (n = 6). Fluorescence minus one (FMO) controls are shown in gray. **(G**) Bar graphs summarize the MFI±SD of CCR2, CCR4, CCR6 and CD86 (n = 4–6) in cells left unexposed (white), exposed to PUUV (ocean blue) or UV PUUV (patterned ocean blue). **(H**) Flow cytometry dot plots show changes in CCR7 in cells exposed to PUUV or unexposed. Numbers in gate depict the frequency of CCR7^+^ cells out of total CM (n = 4) or CD1c^+^ MDC (n = 6). (**I**) Bar graphs summarize the MFI±SD of CCR7 (n = 4–6). Statistical differences were assessed using paired *t*-test: * *p*<0.05, n.s. not significant.

Finally, we established an experimental system to address the loss of MNPs in blood of patients with acute HFRS. Classical monocytes and CD1c^+^ MDCs were isolated from blood of healthy volunteers (Fig 7A) and exposed to PUUV or UV-inactivated PUUV *in vitro*. We measured the frequency of cells expressing PUUV antigens over time by immunofluorescence staining using human anti-PUUV serum (Fig 7B). Forty hours post infection, 1.4% of classical monocytes and 0.4% of CD1c^+^ MDCs were infected by PUUV (Fig 7C), whereas PUUV antigen was undetectable in cells that were either uninfected or exposed to UV-inactivated PUUV. In addition to detecting viral proteins, PUUV RNA was detected by qRT-PCR in classical monocytes and CD1c^+^ MDCs exposed to replicating PUUV, at approximately 200-fold higher than in cells exposed to UV-inactivated PUUV (Fig 7D). However, no replicating viruses were detected in supernatants of infected cells, suggesting that PUUV replication is restricted in these cells (S4 Fig). Neither replicating nor UV-inactivated PUUV decreased the viability of these cells compared to uninfected cells (Fig 7E), typical of the non-cytopathic effects of hantavirus [1]. Interestingly, exposure to PUUV improved the viability of classical monocytes significantly at 40 hours (*p*<0.01) and 60 hours (*p*<0.001) (Fig 7E). When exposed to Hantaan virus (HTNV) that causes severe HFRS, we similarly observed that classical monocytes and CD1c^+^ MDCs were susceptible to infection *in vitro*, without the induction of cell death (S5 Fig).

Both classical monocytes and CD1c^+^ MDCs also responded to PUUV exposure by modulating their expression of chemokine receptors over time (S6 Fig). CCR2, important for mobilization of monocytes from bone marrow to peripheral tissues [22, 53], was downregulated on both classical monocytes (*p*<0.05) and CD1c^+^ MDCs after 40 hours of PUUV exposure (*p*<0.05) compared to uninfected controls (Fig 7F and 7G). CCR4 and CCR6, chemokine receptors that have been associated with tissue homing, were upregulated on classical monocytes (*p*<0.05) and CD1c^+^ MDCs respectively after 12 hours of PUUV exposure compared to cells that were not exposed to virus (Fig 7F and 7G). Both classical monocytes and CD1c^+^ MDCs (*p*<0.05) also responded to PUUV exposure by upregulating the migratory chemokine receptor CCR7 typically expressed by mature cells (Fig 7H and 7I). In line with their increased CCR7 expression, both classical monocytes and CD1c^+^ MDCs also upregulated the co-stimulatory molecule CD86 (*p*<0.05) upon exposure to replicating PUUV, but not to UV-inactivated PUUV (Fig 7F and 7G). In summary, both classical monocytes and CD1c^+^ MDCs were susceptible to PUUV infection *in vitro*, but infection did not result in cell death. The observed regulation of chemokine receptor expression on classical monocytes and CD1c^+^ MDCs upon exposure to PUUV *in vitro* provides a platform for further investigations into the molecular mechanisms governing the redistribution of MNPs observed in patients with acute HFRS.

## Discussion

In this study, we explored the involvement of monocytes and DCs during hantavirus infection by characterizing MNPs at the initial site of infection: the airways, where virions enter their human host upon inhalation of aerosolized hantavirus-containing rodent excreta. Viral RNA, as previously shown [14], can be detected in BAL cells of patients during acute HFRS. An expansion of cytotoxic CD8^+^ T cells in BAL has been shown to correlate with disease severity [14]. In addition to CD8^+^ T cells lining the airways as reflected by BAL sampling, we now demonstrate the presence of CD8^+^ T cells in the bronchial tissue of hantavirus-infected patients. In the same HFRS patients, high levels of HLA-DR^+^ or CD11c^+^ cells were observed in the bronchial biopsies, suggesting an influx of monocytes and/or MDCs. An increase in the number of CD123^+^ cells also suggests an infiltration of PDCs into the airways. The presence of monocytes and DCs in the airways might explain the observed increase in CD8^+^ T cells present in the airways during acute HFRS. During influenza virus infection in mice, recruitment of DCs to the lungs is necessary for mounting adaptive immune responses needed for efficient viral clearance [33–35]. Specifically, local interaction in the lungs between antigen-bearing DCs is required for protective CD8^+^ T cell responses [54]. A careful investigation of how the MNPs interact with CD8^+^ T cells in the airways of hantavirus-infected patients would facilitate understanding of whether MNPs contribute to pathogenesis or immunity, by activating or controlling CTL activity.

As hantavirus infection is systemic [3], we also characterized the absolute numbers of six distinct MNP populations in the blood of patients over the course of disease, from acute infection to convalescence. We found a depletion of all populations, especially MDCs, in the peripheral blood of PUUV-infected patients during the acute phase of HFRS, coinciding with the presence of viral RNA in blood. During acute HFRS, CCR7 was upregulated on several monocyte and DC populations, indicating a mobilization of cells from the blood toward lymph nodes or peripheral tissues. *In vitro*, our data further demonstrated that classical monocytes and CD1c^+^ MDCs were susceptible to PUUV infection and remained alive. Although Markotic *et al*. suggested a differentiation of monocytes into DC-like cells after hantavirus infection *in vitro* [55], we were not able to identify monocytes expressing DC markers by flow cytometry upon PUUV exposure. Our data corroborated earlier findings by Raftery *et al*. and Temonen *et al*. suggesting that human DCs and monocytes may contribute to pathogenesis: both monocytes and DCs remain alive after infection, potentially leading to viral dissemination due to their migratory properties [56, 57].

Hantavirus infection is not marked by a general loss of immune cells or leukopenia in circulation of patients, since these viruses do not cause obvious cytopathic effects [56, 58]. Instead, the numbers of NK cells in blood are expanded in PUUV-infected patients [11, 13, 59]. We anticipated that a similar expansion of MNPs would be detected in blood from our patients as has been reported in those with other acute viral infections, as both monocytes and DCs are mobilized from the bone marrow to partake in the innate response to a viral infection [27, 45, 46, 60, 61]. In contrast, we found a depletion of all monocyte and DC subsets in blood during acute HFRS. Although Tang *et al*. reported an expansion of intermediate monocytes in the blood of HFRS patients [62], no such increase was observed in the present study. The cause of this disparity could be technical due to our gating strategy that excluded all lineage^+^ and HLA-DR^-^ cells. Alternatively, biological differences between the virus strains could yield differing results, as their patient cohort was infected with HTNV whereas our patients were infected with PUUV, the endemic hantavirus strain in Sweden. In line with our findings, the loss of DCs has been documented in patients with acute influenza A virus (IAV) and human immunodeficiency virus (HIV) infections, related to depletion and impaired function of DCs during acute infection [63–66].

While all DC populations in peripheral blood returned to normal values during the convalescent phase of HFRS, we noted that the absolute numbers of intermediate monocytes and non-classical monocytes remained low, even more than 100 days after onset of disease (S3 Table). Monocytes arise from bone marrow precursors, differentiating from classical monocytes via intermediate monocytes to non-classical monocytes in their lifetime [67, 68]. We speculate that in PUUV-infected patients, there may be a delay in the developmental progression of circulating classical monocytes, even during convalescence. The prolonged expansion of NK cells in the circulation of HFRS patients [11] could provide a source of IFN gamma, a cytokine known to activate classical monocytes to a more inflammatory phenotype [69]. Additionally, the emerging concept of trained immunity [70] suggests that monocyte precursors in the bone marrow may be epigenetically modified upon exposure to hantavirus such that they remain poised for future infections.

Our data from experiments performed *in vitro* suggest that even if blood MNPs were susceptible to the virus, hantavirus infection did not lead to cell death. A potential explanation for the stark depletion of DCs observed in blood could be that these cells have migrated out of circulation. The chemokine receptor CCR7 controls the homing of DCs to lymph nodes, where priming of T cells and initiation of adaptive immune responses can occur [49, 50, 71, 72]. Increased CCR7 expression on blood CD1c^+^ MDCs during acute HFRS could indicate that these cells have migrated to the lymph nodes, in response to either direct viral infection, as we could show *in vitro*, or to pro-inflammatory cytokines in serum of patients [73]. Specifically for hantavirus infections, activation of CD4^+^ T cells have been shown to be instrumental in viral control and improved clinical outcome [74].

By infecting monocytes and DCs *in vitro*, we have developed a platform for further dissection of the underlying mechanisms by which exposure to hantavirus determines cellular trafficking. For instance, the chemokine receptor expression may indicate where blood MNPs traffic to during acute HFRS. Accumulation of monocytes in the brain during West Nile virus infection has been related to expression of CCR2, which is important for the egress of monocytes from the bone marrow into tissue [60]. Detection of hantavirus RNA in the bone marrow of a patient [75] suggests that hantavirus could impede the release of monocytes into the bloodstream by downregulating expression of CCR2, as our data suggest. In other respiratory diseases such as chronic obstructive pulmonary disease (COPD), the increased presence of DCs in the lungs correlated with the upregulation of CCR6 on DCs and an increase of the CCR6 ligand (CCL20) in the airways [76]. In mice, expression of CCR4 on T cells imprints them to home to the lungs upon influenza infection [77]. However, these scenarios have not been carefully investigated in the homing of monocytes and DCs into the lungs during viral infection in humans.

In conclusion, blood monocytes and DCs were dramatically depleted during the acute phase of HFRS caused by PUUV. The high numbers of CD8^+^ T cells in the airways [14, 16], correlating with respiratory symptoms experienced by patients, may have been promoted by an infiltration of MNPs into the airways, as demonstrated in bronchial biopsies of hantavirus-infected patients in this study. As the viral load subsides in the blood, the numbers of blood monocytes and DCs also return to normal values. By establishing *in vitro* hantavirus infections of MNPs, the descriptive nature of patient data can be complemented in future *in vitro* studies to elucidate the mechanisms of how hantavirus infection can orchestrate the mobilization of monocytes and DCs from the blood into peripheral tissues such as the respiratory tract, and lymphoid organs. A better understanding on the role of monocytes and DCs during hantavirus infection is valuable in the development of immunomodulatory strategies to treat hantavirus-infected patients.

## Materials and methods

### Ethics statement

The study protocol was approved by the regional Ethical Review Board at Umeå University, Umeå, Sweden. Written informed consent was obtained from study subjects, all of whom were adults.

### Patient samples

Peripheral blood, bronchoalveolar lavage (BAL) and endobronchial biopsies were prospectively obtained from 17 hospitalized PUUV-infected patients between 2008 and 2011. The criteria for study enrollment were described earlier [14]. Peripheral blood samples for flow cytometry analysis were collected during the acute phase (2–14 days after disease onset; median 6 days). Follow-up samples were taken throughout the first weeks of infection as well as the convalescent phase (>15 days after disease onset). Patients were monitored using qRT-PCR to assess plasma viral load until two consecutive measurements were negative (median 11 days) [78]. Briefly, viral RNA was extracted from plasma and cDNA was generated. qRT-PCR was performed in triplicate using primers designed based on PUUV RNA sequences. No fatal cases were observed in this study cohort. Twelve uninfected age- and sex-matched blood donors were included in this study. They underwent bronchoscopy for the collection of endobronchial biopsies and bronchoalveolar lavage (BAL) as well as peripheral blood draw. Standard clinical procedures, including differential cell counts, were used to obtain clinical data for all subjects used in this study (S1 Table) [79].

### Bronchoscopy

Bronchoscopy was performed on all patients 6 to 14 days (median 9 days) after onset of symptoms. Patients underwent bronchoscopy as soon as their clinical condition allowed them to withstand the procedure. This included absence of hypotension or hypoxemia as well as improvements of coagulation parameters to avoid bleeding. Bronchoscopy was performed as soon as possible and when blood platelet count was higher than 100 x 10^9^. At that time, all patients were still in need of hospital care due to the acute infection. In brief, patients and UCs were treated with oral midazolam (4–8 mg) and intravenous glycopyrronium (0.2–0.4 mg) 30 minutes (min) before the bronchoscopy. For topical anesthesia, lidocaine was applied, and additional lidocaine was administered in the larynx and bronchi during the procedure. A flexible video bronchoscope (Olympus BF IT200) was inserted through the mouth via a plastic mouthpiece. From each patient, four to six endobronchial biopsies were taken from the main carina and the main bronchial divisions on the left side using fenestrated forceps (Olympus FB-21C). BAL was obtained with saline solution (3 x 60 mL) from the contralateral side. BAL samples were filtered through a 100 μm nylon filter (Syntab) and centrifuged at 400 x g for 15 min at 4°C.

### Biopsy processing and quantification of cells by immunohistochemistry

Endobronchial biopsies were processed and embedded into glycol methacrylate resin (Polyscience), as previously described [80]. Sections from biopsies (2 μm) were stained in duplicates with anti-CD8, HLA-DR, CD11c, and CD123 (all BD Biosciences) followed by the rabbit anti-mouse (Dako) biotinylated secondary antibody. The immunostaining was performed as previously described [13]. All sections were visualized with 3-amino-9-ethylcarbazole (AEC), and cell nuclei were counterstained with Mayer hematoxylin (Histo Lab). Finally, all sections were analyzed using a high-resolution digital scanner, NanoZoomer-XR (HAMAMATSU) to convert them into digital images. A blinded analysis was performed using the scanned sections and NanoZoomer Digitial Pathway View2 software (NDP View; HAMAMATSU). The number of positive cells was expressed as cells/mm and cells/mm^2^ of epithelium and lamina propria, respectively. Quantification of HLA-DR molecules was carried out with a Leica DMR-X microscope (Leica Microsystems GmbH) coupled to computerized image analysis (Leica Qwin 5501W; Leica Imaging Systems) as described previously [81].

### Peripheral blood processing

For isolation of peripheral blood mononuclear cells (PBMCs), whole blood from PUUV-infected patients and UC was collected in CPT tubes (BD) and centrifuged according to manufacturer’s instructions. The separated suspension of PBMCs was harvested and then washed in PBS. PBMCs were frozen in 90% human albumin (Octapharma), 10% DMSO (WAKO-Chemie Medical), and 50 IE heparin (LEO Pharma), and stored in liquid nitrogen for later analysis. Absolute numbers of all monocyte and DC subsets were calculated by using the absolute lymphocyte and monocyte counts obtained on the automated hematology analyzer and the percentages of events in each respective gate obtained from flow cytometry data.

### Isolation of monocytes and DCs

Monocytes and primary CD1c^+^ MDCs were isolated from buffy coats obtained from Karolinska University Hospital (Stockholm, Sweden) as previously described [82]. Monocytes were isolated using the human monocyte enrichment kit (RosetteSep; StemCell Technologies) according to the manufacturer’s instructions. The blood was diluted, carefully layered on Ficoll-Paque PLUS (GE Healthcare Biosciences) and centrifuged for 20 min at 1800 x g at room temperature. For isolation of human CD1c^+^ MDCs, magnetic labeling using CD1c^+^ MDC isolation kit (Miltenyi Biotec) was used on enriched populations of monocytes. Monocytes and MDCs were cultured in RPMI1640 (Sigma-Aldrich) with 10% fetal bovine serum (FBS), 1% penicillin/streptomycin and 1% L-Glutamine (all Invitrogen). Cells were cultured at 1x10^6^ cells per mL of complete medium. MDCs were additionally supplemented with 2 ng/mL GM-CSF (R&D Systems).

### Hantavirus infection of human DCs and monocytes *in vitro*

PUUV strain Kazan and HTNV strain 76–118 were propagated on Vero E6 cells (ATCC Vero C1008) as previously described [48]. The virus stocks were titrated on Vero E6 cells for calculations of multiplicity of infection (MOI). UV inactivation of hantaviruses was performed for 25 seconds using a VL215G Vilber Lourmat UV lamp (Torcy), as a negative control for productive infection. Cells were exposed to medium alone (uninfected), infected with hantaviruses or exposed to UV-inactivated hantaviruses at an MOI of 7.5 for 2 hours (h). Cells were then washed and incubated for 12–60 h at 37°C. Supernatants were collected after centrifugation and were stored at −80°C until further analysis. Cells were stained for flow cytometric analysis.

### Flow cytometric analysis

Cell suspensions were stained with Live/Dead Aqua fixable dead cell stain kit (Invitrogen) to exclude dead cells. Non-specific binding was prevented by adding FcR blocking reagent (Miltenyi Biotec) followed by surface staining with conjugated Abs (S5 Table). Briefly, cells were stained for 15 min at 4°C in FACS buffer (PBS with 2% fetal bovine serum) and fixed in 1% paraformaldehyde (PFA). For chemokine receptor staining, cells were stained for 15 min at 37°C prior to addition of cell surface antibodies for another 15 min at RT. For HTNV-infected cells, intracellular staining with anti-nucleocapsid protein (N) antibody (B5D9, Progen) was assessed using a standard protocol. In brief, cells were stained for surface markers, fixed and permeabilized using Transcription Factor Staining Buffer Set (eBioscience). Cells were analyzed by flow cytometry using a LSRII instrument or LSRFortessa (both BD Biosciences) and data were analyzed using FlowJo X software (Tree Star).

### PUUV RNA analysis of cells infected with PUUV *in vitro*

At 60 h post infection, RNA from DCs and monocytes infected with PUUV *in vitro* was isolated using 450 μl TriPure Isolation Reagent (Roche Diagnostics). The relative levels of PUUV RNA in PUUV-infected DCs and monocytes was assessed using a qRT-PCR assay, as previously described [83]. *β*-actin mRNA levels were measured in parallel, using a commercially available TaqMan gene expression assay (4333762; Applied Biosystems). The expression of PUUV S segment RNA was calculated against the housekeeping gene *β*-actin: 2^-[Ct(PUUV gene)-Ct(B-Actin)]^.

### Immunofluorescence assay of cells infected with PUUV *in vitro*

At 40 h post infection, classical monocytes and CD1c^+^ myeloid DCs were adhered for 20 min on Alcian blue-coated coverslips at 100 000 cells per condition. Cells on the coverslips were gently washed in PBS and fixed with pre-warmed 4% paraformaldehyde for 20 min at room temperature. Cells were then blocked with PBS containing 1% normal goat serum and permeabilized with 0.1% Triton-X 100 (Sigma) and stained with human anti-PUUV serum for 1 hour. Secondary antibodies against human IgG conjugated to Alexa Fluor 488 were used. Additionally, CD1c^+^ MDCs were co-stained with anti-HLA-DR conjugated to Alexa Fluor 647. Coverslips were mounted on glass slides with Prolong Diamond Antifade mountant with DAPI (Molecular Probes). Confocal images were acquired on a Zeiss LSM700 using a 10x objective. PUUV^+^ cells were enumerated out of 1000–2000 cells per condition using FIJI ImageJ software (NIH).

### Detection of Granzyme B by enzyme-linked immunosorbent assay (ELISA)

Levels of granzyme B in BAL fluid were measured using the commercially available Human Granzyme B ELISA kit (Abcam).

### Statistical analysis

For all patient data generated *ex vivo*, mean cell counts of monocyte and DC populations were modeled using Poisson regression with patient-specific random intercept and robust standard errors. The proportions of CCR7^+^ CMs, IM, NCM, CD1c^+^ MDCs, CD141^+^ MDCs, and PDCs were modeled using logistic regression with patient-specific random intercept. Random intercepts were used to account for the potential dependence among repeated blood measurements over time. Number of days since symptoms' onset in HFRS patients was the predictor of interest and was categorized as acute phase (2–14 days) or convalescent phase (>15 days). UC served as the reference group. Correlations were analyzed using Spearman’s rank correlation coefficient. For *in vitro* experiments, statistical significance was assessed using paired *t*-test. Comparisons for IHC data are by Mann–Whitney U-test. Data were analyzed using GraphPad Prism version 6.0 (GraphPad Software) and Stata version 14.1 (StataCorp, College Station, TX). All the reported *p*-values are two-sided and *p*-values<0.05 was considered statistically significant.

## Supporting information

S1 Table Clinical and laboratory characteristics of HFRS patients. (DOCX)

S2 Table Distribution of peripheral blood sampling in HFRS patients over time. (DOCX)

S3 Table Average numbers of blood mononuclear phagocytes during acute and convalescent HFRS. (DOCX)

S4 Table Statistical analysis of blood mononuclear phagocytes during acute and convalescent HFRS. (DOCX)

S5 Table Antibodies. (DOCX)

S1 Fig Detectable viral load in plasma of HFRS patients measured by real time reverse transcriptase polymerase chain reaction (RT-PCR) of PUUV RNA. Graphs show detectable viral load in the earliest clinical sample available, as measured by quantification of PUUV RNA in plasma from each HFRS patient. Viral load was monitored until patients were negative in two successive measurements. Matched samples from individual patients are indicated with dotted lines. Statistically significant differences were assessed using paired *t*-test.
(DOCX)

S2 Fig Identification of DC subsets and monocytes in PBMCs from HFRS patients and uninfected controls. Gating strategy used to identify monocytes as well as MDC and PDC subsets within the live lin^-^ HLA-DR^+^ fraction in blood PBMCs.
(DOCX)

S3 Fig Reproducibility of DC frequencies with cryopreserved PBMCs in HFRS patients and controls. (**A**) No significant differences in the frequency of immune cells in healthy PBMCs analyzed by flow cytometry fresh (black) or after freeze-thawing (white). PBMCs were isolated from buffy coats and all samples were studied fresh directly after the procedure and after cryopreservation, respectively. Representative histograms from one healthy donor are shown. (**B**) No significant differences between fresh (black, n = 2) and frozen (white, n = 9) cells in the frequencies of MNPs of total cells in PBMCs from acute (2–7 days after onset if the disease) HFRS patients. Bar graphs show mean±SD. Statistically significant differences were assessed using unpaired *t*-test.
(DOCX)

S4 Fig Abortive replication of PUUV in monocytes and CD1c^+^ MDCs exposed to PUUV over time. Graph shows declining levels of remaining input virus measured as focus forming units (FFU) per mL in the supernatants of monocytes (green) and CD1c^+^ MDCs (coral) after 12, 20, 40 and 60 hours of infection with PUUV.
(DOCX)

S5 Fig Susceptibility of classical monocytes and CD1c^+^ MDCs to HTNV infection *in vitro*. (**A**) Human classical monocytes (CM) and CD1c^+^ MDCs were isolated from peripheral blood of healthy volunteers. Cells were left unexposed, exposed to HTNV or UV-inactivated HTNV for 2 h at an MOI of 7.5. Cells were washed and subsequently incubated for 12–60 h. Flow cytometry dot plots show live, HLA-DR^+^ CD11c^+^ CD14^+^ CD16^-^ classical monocytes (left panel) or live, CD11c^+^ CD1c^+^ MDCs (right panel). Numbers in gate depict the frequency of HTNV^+^ cells out of total live cells. One representative donor is shown. **(B)** Bar graphs summarize the mean±SD frequency of HTNV^+^ cells as assessed by flow cytometry in CM (left panel, n = 4) and CD1c^+^ MDCs (right panel, n = 3) left unexposed (white), exposed to HTNV (purple) or UV HTNV (patterned purple). **(C)** Viability of cells at 24 hours was assessed by flow cytometry based on a LIVE/DEAD dye. Graphs show mean±SD viability of CM (left panel, n = 4) or CD1c^+^ MDC (right panel, n = 3).
(DOCX)

S6 Fig Dynamic changes in expression of chemokine receptors on classical monocytes and CD1c^+^ MDCs upon exposure to PUUV *in vitro*. (**A**) Human CM and CD1c^+^ MDCs were isolated from peripheral blood of healthy volunteers. Cells were left unexposed (white), exposed to PUUV (ocean blue) or UV PUUV (patterned ocean blue) for 2 h at an MOI of 7.5, 1 or 0.1. Excess viruses were removed and cells were subsequently infected for 12–60 h. Viability of cells at 24 h was assessed by flow cytometry based on a LIVE/DEAD dye. Graphs show mean±SD viability of CM (left panel, n = 3) or CD1c^+^ MDC (right panel, n = 3). (**B**) Bar graphs summarize the MFI±SD of CCR2, CCR4, CCR6, CCR7 and CD86 (n = 4–6).
(DOCX)
