# Supplementary material for: Human hantavirus infection elicits pronounced redistribution of mononuclear phagocytes in peripheral blood and airways
Source: PLoS Pathog. 2017 Jun 22;13(6):e1006462. doi: 10.1371/journal.ppat.1006462 (PMC5498053; doi:10.1371/journal.ppat.1006462)
Supplement: S1 Table — (DOCX) [file ppat.1006462.s001.docx]

**Table S1. Clinical and laboratory characteristics of HFRS patients.**

| Patient No. | Sex | Age [years] | Smoker | Days in Hospital | Respiratory Symptoms* | Oxygen Treatment | Creatinine  [µmol/L]^§^ | | Platelet count  [10^9^/L]^¶^ | | WBC  [10^9^/L]^#^ | | CRP  [mg/L] ^†^ | |
| --- | --- | --- | --- | --- | --- | --- | --- | --- | --- | --- | --- | --- | --- | --- |
|  |  |  |  |  |  |  | Acute | Conv. | Acute | Conv. | Acute | Conv. | Acute | Conv. |
| 1 | F | 31 | No | 5 | Yes | No | 173 | 56 | 66 | 200 | 9,1 | 10,9 | 36 | 13 |
| 2 | F | 45 | Yes | 5 | No | No | 108 | 62 | 79 | 213 | 6,9 | 5,6 | 76 | <3 |
| 3 | F | 49 | No | 6 | No | No | 234 | 48 | 27 | 322 | 27,0 | 4,7 | 133 | <3 |
| 4 | F | 50 | Yes | 6 | Yes | No | 75 | 49 | 94 | 333 | 7,0 | 3,8 | 35 | <3 |
| 5 | F | 53 | No | 2 | Yes | No | 120 | 61 | 305 | 284 | 12,1 | 5,3 | 36 | <3 |
| 6 | F | 54 | Yes | 4 | Yes | No | 59 | 41 | 41 | 140 | 7,7 | 5,3 | 78 | 5 |
| 7 | F | 61 | Yes | 6 | No | No | 161 | 65 | 44 | 247 | 8,8 | 8,0 | 123 | <3 |
| 8 | F | 64 | No | 8 | Yes | No | 62 | 56 | 59 | 211 | 7,0 | 4,8 | 69 | <3 |
| 9 | F | 65 | Yes | 5 | No | Yes | 276 | 62 | 89 | 233 | 15,4 | 7,3 | 47 | <3 |
| 10 | F | 68 | Yes | 4 | Yes | Yes | 276 | 91 | 63 | 340 | 8,2 | 7,8 | 187 | 7 |
| 11 | F | 69 | No | 9 | No | No | 138 | 57 | 18 | 200 | 9,6 | 6,5 | 130 | <3 |
| 12 | M | 51 | No | 3 | Yes | No | 121 | 83 | 55 | 262 | 8,4 | 6,5 | 38 | <3 |
| 13 | M | 54 | Yes | 4 | Yes | No | 391 | 89 | 70 | 244 | 9,0 | 5,4 | 249 | <3 |
| 14 | M | 54 | No | 6 | Yes | Yes | 186 | 88 | 81 | 346 | 15,1 | 9,0 | 82 | <3 |
| 15 | M | 60 | No | 5 | No | No | 1072 | 93 | 129 | 237 | 5,3 | 5,0 | 67 | 6 |
| 16 | M | 62 | No | 9 | Yes | Yes | 377 | 87 | 25 | 172 | 16,1 | 8,8 | 206 | <3 |
| 17 | M | 67 | No | 5 | No | Yes | 185 | 97 | 58 | 226 | 9,9 | 6,2 | 199 | <3 |

F=female, M=male. Acute phase laboratory results represent highest values for creatinine level, WBC and CRP, and lowest values for platelet count. Laboratory findings normalized in convalescent (conv.) phase after 3 months (p<0.001 for all, Wilcoxon signed-ranks test).

*Patients experiencing respiratory symptoms (n=10), dry cough (n=5) and dyspnea (n=8).

^§^Plasma creatinine; reference <90 µmol/L for women, <105 µmol/L for men; patient median maximum value during acute phase 173 µmol/L.

^¶^Platelet count; normal range 165-387 x10^9^/L for women, 145-348 x10^9^/L for men; patient median nadir value during acute phase 63 x10^9^/L.

^#^White blood cell (WBC) count; normal range 3.5-8.8 x10^9^/L, patient median maximum value during acute phase 9.0 x10^9^/L.

^†^Plasma C-reactive protein (CRP); reference <3 mg/L; patient median maximum value during acute phase 78 mg/ml.
