# Supplementary material for: Human hantavirus infection elicits pronounced redistribution of mononuclear phagocytes in peripheral blood and airways
Source: PLoS Pathog. 2017 Jun 22;13(6):e1006462. doi: 10.1371/journal.ppat.1006462 (PMC5498053; doi:10.1371/journal.ppat.1006462)
Supplement: S3 Table — (DOCX) [file ppat.1006462.s003.docx]

**Table S3. Average numbers of blood mononuclear phagocytes during acute and convalescent HFRS.**

| Absolute numbers of cells per µL of blood | UC | | Acute HFRS | | | | | | Convalescent HFRS | | | | | |
| --- | --- | --- | --- | --- | --- | --- | --- | --- | --- | --- | --- | --- | --- | --- |
|  |  |  | 2-7 | | 8-10 | | 11-14 | | 15-21 | | 40-99 | | >100 | |
|  |  |  | Days after onset of HFRS | | | | | | | | | | | |
| Classical monocytes | 393,70 | ±144 | 117,80 | ±200 | 274,60 | ±260 | 158,00 | ±264 | 412,00 | ±283 | 277,80 | ±193 | 449,80 | ±291 |
| Intermediate monocytes | 33,76 | ±15 | 6,60 | ±8 | 17,84 | ±22 | 6,44 | ±9 | 22,38 | ±18 | 24,05 | ±27 | 17,88 | ±12 |
| Non-classical monocytes | 43,36 | ±24 | 2,65 | ±3 | 12,68 | ±11 | 9,50 | ±10 | 35,70 | ±39 | 37,60 | ±52 | 26,94 | ±21 |
| CD1c^+^ MDCs | 16,58 | ±6 | 1,43 | ±1 | 7,43 | ±6 | 13,04 | ±12 | 17,63 | ±18 | 18,53 | ±13 | 17,79 | ±12 |
| CD141^+^ MDCs | 1,10 | ±0,4 | 0,14 | ±0,2 | 0,15 | ±0,1 | 0,35 | ±0,4 | 0,44 | ±0,5 | 0,62 | ±0,8 | 0,99 | ±0,8 |
| PDCs | 10,09 | ±8 | 3,15 | ±3 | 3,87 | ±2 | 4,61 | ±4 | 5,52 | ±2 | 11,54 | ±9 | 12,54 | ±8 |
| *n* | 12 | | 9 | | 9 | | 10 | | 4 | | 10 | | 16 | |

Values are mean ± standard deviation. *n* = number of samples. Uninfected controls (UC).
