# Supplementary material for: Human hantavirus infection elicits pronounced redistribution of mononuclear phagocytes in peripheral blood and airways
Source: PLoS Pathog. 2017 Jun 22;13(6):e1006462. doi: 10.1371/journal.ppat.1006462 (PMC5498053; doi:10.1371/journal.ppat.1006462)
Supplement: S4 Table — (DOCX) [file ppat.1006462.s004.docx]

**Table S4. Statistical analysis of blood mononuclear phagocytes during acute and convalescent HFRS.**

| **Relative change in mean cell count (*p*-value)**  (Time modeled as two periods: acute [days 2-14] and convalescent [days 15-max]) | | | |
| --- | --- | --- | --- |
| Cell type | UC  vs  Acute HFRS | UC  vs  Convalescent HFRS | Acute HFRS  vs  Convalescent HFRS |
| Classical monocytes | <0,001 | 0,244 | <0,001 |
| Intermediate monocytes | 0,001 | 0,016 | 0,403 |
| Non-classical monocytes | <0,001 | 0,058 | 0,001 |
| CD1c^+^ MDCs | 0,002 | 0,702 | 0,003 |
| CD141^+^ MDCs | <0,001 | 0,217 | <0,001 |
| PDCs | 0,001 | 0,484 | <0,001 |
|  | | | |
| **Relative change** **in mean cell count (%)**  (Time modeled as a continuous variable) | | | |
| Cell type | UC  vs  day 2 | Acute HFRS^#^  [days 2-14] | Convalescent HFRS^#^  [days 15-max] |
| Classical monocytes | -80,3 | +10,8 | +0,1 |
| Intermediate monocytes | -72,7 | +5,7 | -0,1 |
| Non-classical monocytes | -96,5 | +26,6 | -0,1 |
| CD1c^+^ MDCs | -94,2 | +27,7 | -0,1 |
| CD141^+^ MDCs | -97,3 | +30,3 | +0,0 |
| PDCs | -85,6 | +16,3 | +0,2 |

^#^For every 1-day increase.

Uninfected controls (UC).
