# Supplementary material for: Human hantavirus infection elicits pronounced redistribution of mononuclear phagocytes in peripheral blood and airways
Source: PLoS Pathog. 2017 Jun 22;13(6):e1006462. doi: 10.1371/journal.ppat.1006462 (PMC5498053; doi:10.1371/journal.ppat.1006462)
Supplement: S5 Table — (DOCX) [file ppat.1006462.s005.docx]

**Table S5. Antibodies.**

| Antigen | Fluorochrome | Clone | Company |
| --- | --- | --- | --- |
|  |  |  |  |
| CCR2 | BV605 | K036C2 | BioLegend |
| CCR4 | PE | L291H4 | BioLegend |
| CCR6 | BV786 | 11A9 | BD Bioscience |
| CCR7 (CD197) | BV711 | G043H7 | BioLegend |
| CCR7 (CD197) | PE | 150503 | R&D |
| CD1c (BDCA-1) | FITC | AD5-8E7 | Miltenyi Biotech |
| CD3 | APC | SK7 | BD Bioscience |
| CD3 | APC-Cy7 | SK7 | BD Bioscience |
| CD4 | FITC | RPA-T4 | BD Bioscience |
| CD8 | PE | SK1 | BD Bioscience |
| CD11c | V450 | B-Ly6 | BD Bioscience |
| CD14 | PE-Cy7 | M5E5 | BD Bioscience |
| CD14 | QD800 | Tuk4 | Invitrogen |
| CD16 | AF700 | 3G8 | BioLegend |
| CD20 | APC-Cy7 | L27 | BD Bioscience |
| CD56 | Pacific Blue | MEM-188 | BioLegend |
| CD70 | PE-CF594 | Ki-24 | BD Bioscience |
| CD86 | APC | 2331 (FUN-1) | BD Bioscience |
| CD103 | PE-Cy7 | Ber-ACT8 | BioLegend |
| CD123 | PE-Cy7 | custom | BD Bioscience |
| CD141 (BDCA-3) | PE | AD5-14H12 | Miltenyi Biotech |
| CD303 (BDCA-2) | FITC | AC144 | Miltenyi Biotech |
| HLA-DR | PE-Cy5 | G46-6 | BD Bioscience |
| HLA-DR | PE-Texas Red | TU36 | Invitrogen |
