# Supplementary material for: Human hantavirus infection elicits pronounced redistribution of mononuclear phagocytes in peripheral blood and airways
Source: PLoS Pathog. 2017 Jun 22;13(6):e1006462. doi: 10.1371/journal.ppat.1006462 (PMC5498053; doi:10.1371/journal.ppat.1006462)
Supplement: S1 Fig — Graphs show detectable viral load in the earliest clinical sample available, as measured by quantification of PUUV RNA in plasma from each HFRS patient. Viral load was monitored until patients were negative in two successive measurements. Matched samples from individual patients are indicated with dotted lines. Statistically significant differences were assessed using paired t-test. (DOCX) [file ppat.1006462.s006.docx]

**
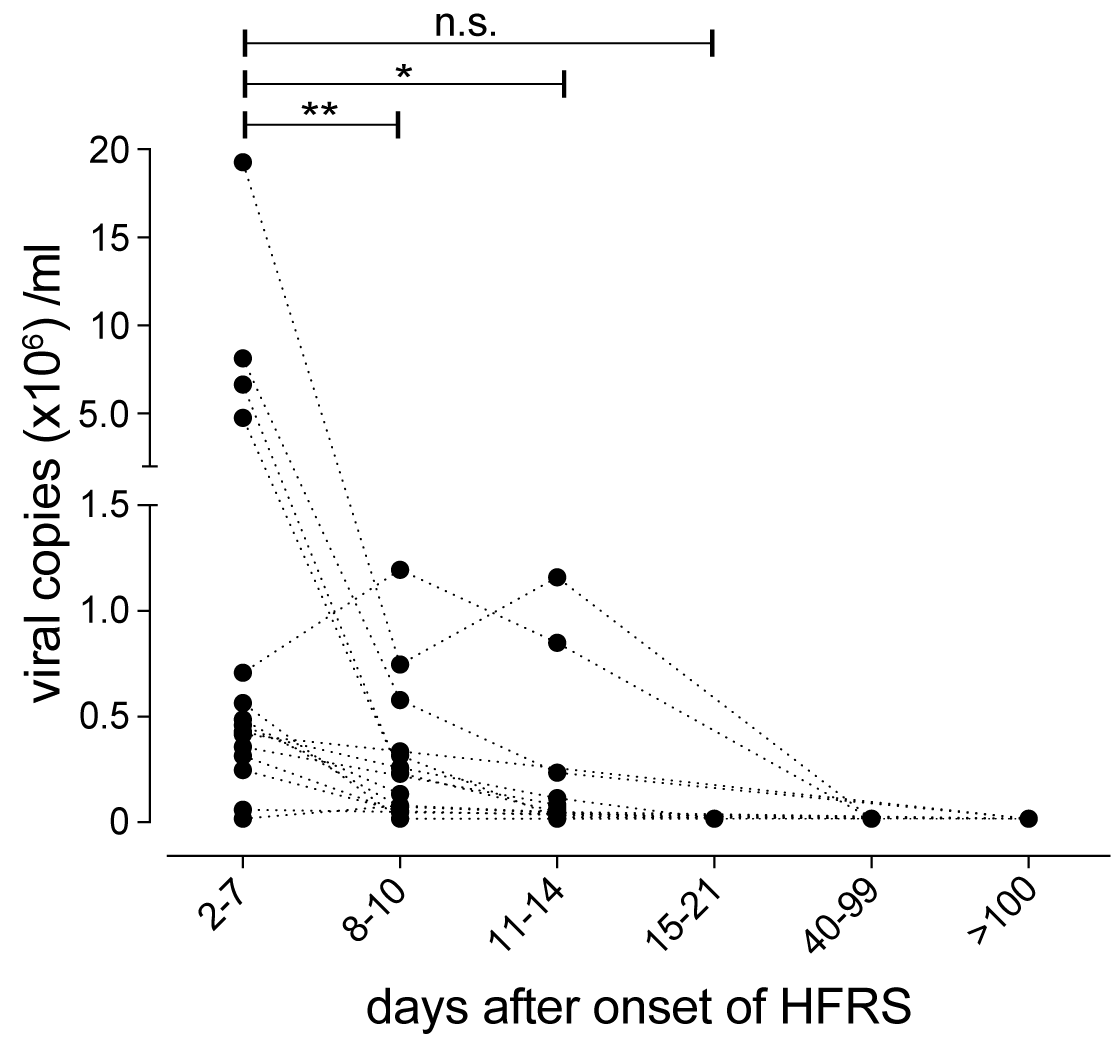
**

**Figure S1. Viral load in plasma of HFRS patients measured by real time reverse transcriptase polymerase chain reaction (RT-PCR) of PUUV RNA.** Graph shows detectable viral load in the earliest clinical sample available, as measured by quantification of PUUV RNA in plasma from each HFRS patient included in this study. Viral load was monitored until patients were negative in two successive measurements. Matched samples from individual patients are indicated with dotted lines. Statistically significant differences were assessed using paired *t*-test.
