# Supplementary material for: Human hantavirus infection elicits pronounced redistribution of mononuclear phagocytes in peripheral blood and airways
Source: PLoS Pathog. 2017 Jun 22;13(6):e1006462. doi: 10.1371/journal.ppat.1006462 (PMC5498053; doi:10.1371/journal.ppat.1006462)
Supplement: S2 Fig — Gating strategy used to identify monocytes as well as MDC and PDC subsets within the live lin- HLA-DR+ fraction in blood PBMCs. (DOCX) [file ppat.1006462.s007.docx]

**
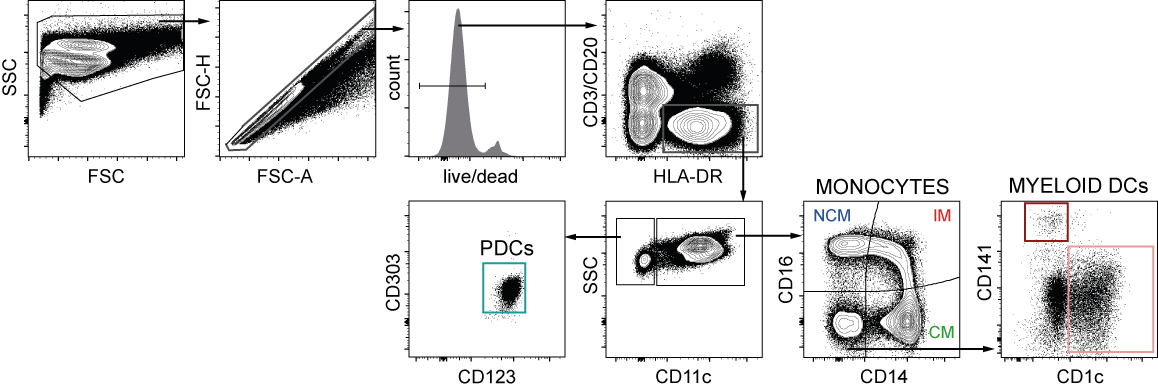
**

**Figure S2. Identification of DC subsets and monocytes in PBMCs from HFRS patients and uninfected controls.** Gating strategy used to identify monocytes as well as MDC and PDC subsets within the live Lin^-^ HLA-DR^+^ fraction in blood PBMCs.
