# Supplementary material for: Human hantavirus infection elicits pronounced redistribution of mononuclear phagocytes in peripheral blood and airways
Source: PLoS Pathog. 2017 Jun 22;13(6):e1006462. doi: 10.1371/journal.ppat.1006462 (PMC5498053; doi:10.1371/journal.ppat.1006462)
Supplement: S3 Fig — (A) No significant differences in the frequency of immune cells in healthy PBMCs analyzed by flow cytometry fresh (black) or after freeze-thawing (white). PBMCs were isolated from buffy coats and all samples were studied fresh directly after the procedure and after cryopreservation, respectively. Representative histograms from one healthy donor are shown. (B) No significant differences between fresh (black, n = 2) and frozen (white, n = 9) cells in the frequencies of MNPs of total cells in PBMCs from acute (2–7 days after onset if the disease) HFRS patients. Bar graphs show mean±SD. Statistically significant differences were assessed using unpaired t-test. (DOCX) [file ppat.1006462.s008.docx]

**
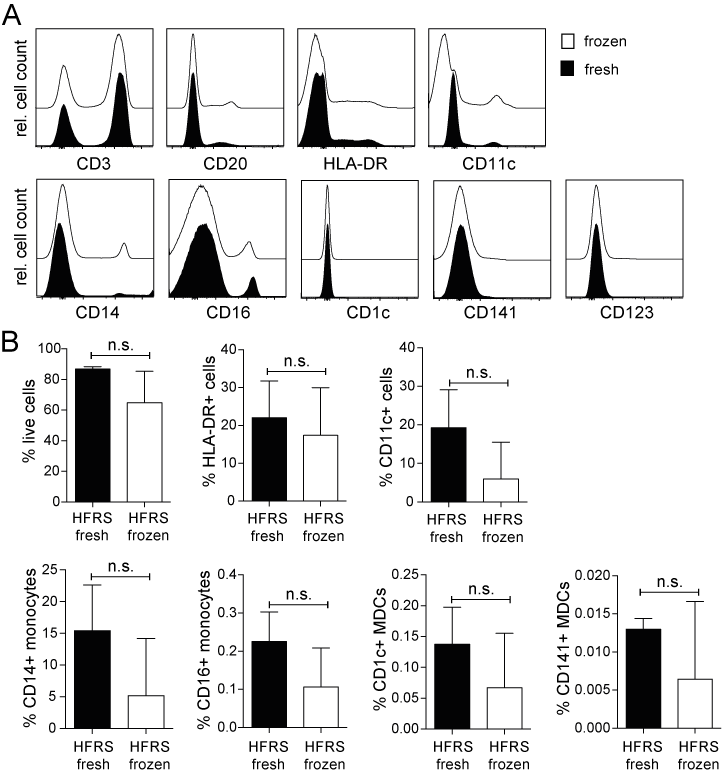
**

**Figure S3. Reproducibility of DC frequencies with cryopreserved PBMCs in HFRS patients and controls.** (**A**) No significant differences in the frequency of immune cells in healthy PBMCs analyzed by flow cytometry fresh (black) or after freeze-thawing (white). PBMCs were isolated from buffy coats and all samples were studied fresh directly after the procedure and after cryopreservation, respectively. Representative histograms from one healthy donor are shown. (**B**) No significant differences between fresh (black, n=2) and frozen (white, n=9) cells in the frequencies of MNPs of total cells in PBMCs from acute (2-7 days after onset if the disease) HFRS patients. Bar graphs show mean±SD. Statistically significant differences were assessed using unpaired *t*-test.
