# Supplementary material for: Human hantavirus infection elicits pronounced redistribution of mononuclear phagocytes in peripheral blood and airways
Source: PLoS Pathog. 2017 Jun 22;13(6):e1006462. doi: 10.1371/journal.ppat.1006462 (PMC5498053; doi:10.1371/journal.ppat.1006462)
Supplement: S4 Fig — Graph shows declining levels of remaining input virus measured as focus forming units (FFU) per mL in the supernatants of monocytes (green) and CD1c+ MDCs (coral) after 12, 20, 40 and 60 hours of infection with PUUV. (DOCX) [file ppat.1006462.s009.docx]

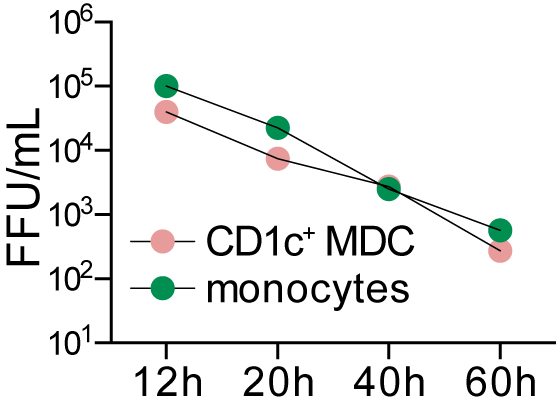


**Figure S4. Abortive replication of PUUV in monocytes and CD1c^+^ MDCs exposed to PUUV over time.** Graph shows declining levels of remaining input virus measured as focus forming units (FFU) per mL in the supernatants of monocytes (green) and CD1c^+^ MDCs (coral) after 12, 20, 40 and 60 hours of infection with PUUV.
