# Supplementary material for: Human hantavirus infection elicits pronounced redistribution of mononuclear phagocytes in peripheral blood and airways
Source: PLoS Pathog. 2017 Jun 22;13(6):e1006462. doi: 10.1371/journal.ppat.1006462 (PMC5498053; doi:10.1371/journal.ppat.1006462)
Supplement: S5 Fig — (A) Human classical monocytes (CM) and CD1c+ MDCs were isolated from peripheral blood of healthy volunteers. Cells were left unexposed, exposed to HTNV or UV-inactivated HTNV for 2 h at an MOI of 7.5. Cells were washed and subsequently incubated for 12–60 h. Flow cytometry dot plots show live, HLA-DR+ CD11c+ CD14+ CD16- classical monocytes (left panel) or live, CD11c+ CD1c+ MDCs (right panel). Numbers in gate depict the frequency of HTNV+ cells out of total live cells. One representative donor is shown. (B) Bar graphs summarize the mean±SD frequency of HTNV+ cells as assessed by flow cytometry in CM (left panel, n = 4) and CD1c+ MDCs (right panel, n = 3) left unexposed (white), exposed to HTNV (purple) or UV HTNV (patterned purple). (C) Viability of cells at 24 hours was assessed by flow cytometry based on a LIVE/DEAD dye. Graphs show mean±SD viability of CM (left panel, n = 4) or CD1c+ MDC (right panel, n = 3). (DOCX) [file ppat.1006462.s010.docx]

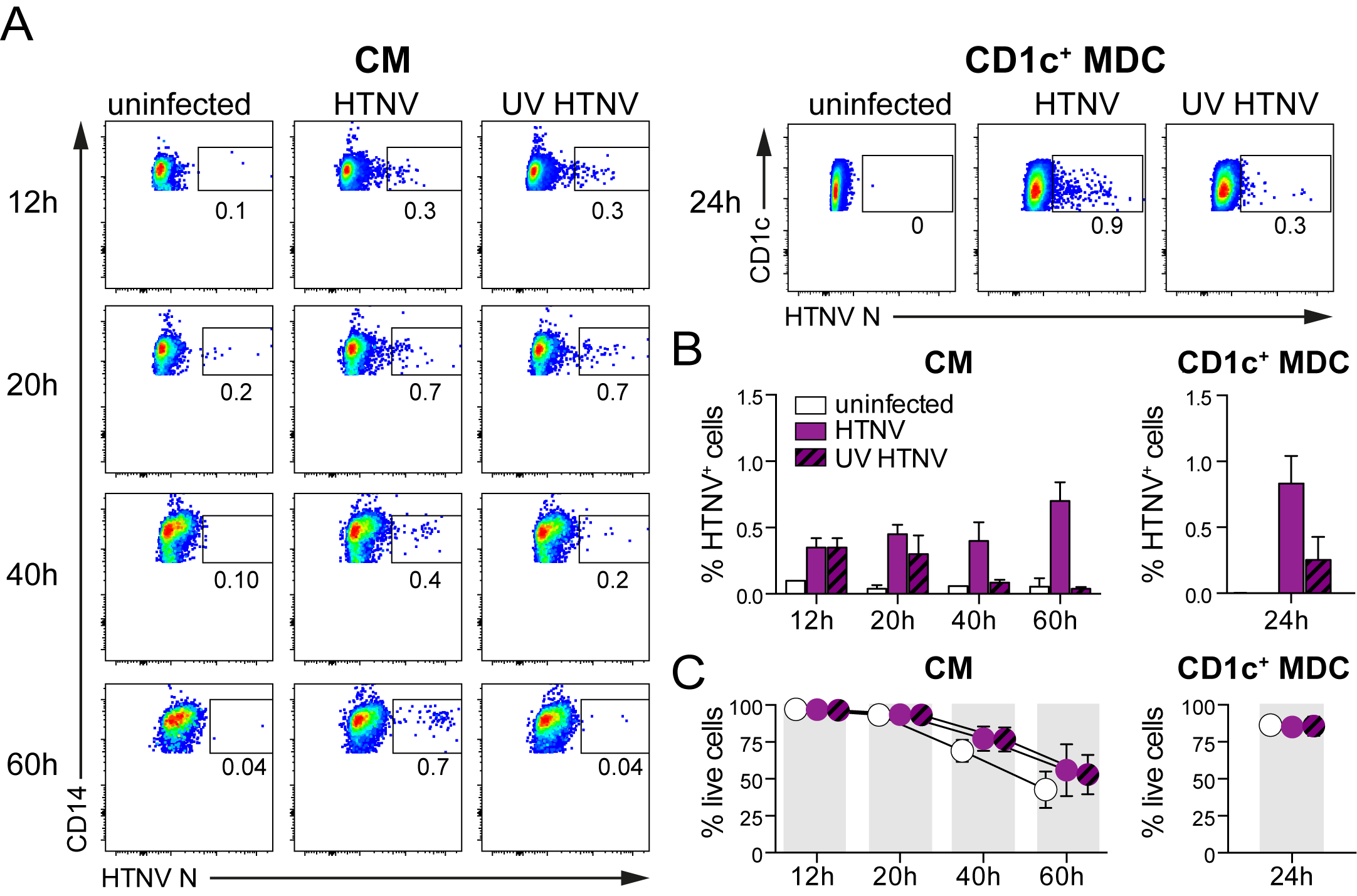


**Figure S5. Susceptibility of classical monocytes and CD1c^+^ MDCs to HTNV infection *in vitro*.** (**A**) Human classical monocytes (CM) and CD1c^+^ MDCs were isolated from peripheral blood of healthy volunteers. Cells were left unexposed, exposed to HTNV or UV-inactivated HTNV for 2 h at an MOI of 7.5. Cells were washed and subsequently incubated for 12–60 h. Flow cytometry dot plots show live, HLA-DR^+^ CD11c^+^ CD14^+^ CD16^-^ classical monocytes (left panel) or live, CD11c^+^ CD1c^+^ MDCs (right panel). Numbers in gate depict the frequency of HTNV^+^ cells out of total live cells. One representative donor is shown. **(B)** Bar graphs summarize the mean±SD frequency of HTNV^+^ cells as assessed by flow cytometry in CM (left panel, n=4) and CD1c^+^ MDCs (right panel, n=3) left unexposed (white), exposed to HTNV (purple) or UV HTNV (patterned purple). **(C)** Viability of cells at 24 hours was assessed by flow cytometry based on a LIVE/DEAD dye. Graphs show mean±SD viability of CM (left panel, n=4) or CD1c^+^ MDC (right panel, n=3).
