# Supplementary material for: Human hantavirus infection elicits pronounced redistribution of mononuclear phagocytes in peripheral blood and airways
Source: PLoS Pathog. 2017 Jun 22;13(6):e1006462. doi: 10.1371/journal.ppat.1006462 (PMC5498053; doi:10.1371/journal.ppat.1006462)
Supplement: S6 Fig — (A) Human CM and CD1c+ MDCs were isolated from peripheral blood of healthy volunteers. Cells were left unexposed (white), exposed to PUUV (ocean blue) or UV PUUV (patterned ocean blue) for 2 h at an MOI of 7.5, 1 or 0.1. Excess viruses were removed and cells were subsequently infected for 12–60 h. Viability of cells at 24 h was assessed by flow cytometry based on a LIVE/DEAD dye. Graphs show mean±SD viability of CM (left panel, n = 3) or CD1c+ MDC (right panel, n = 3). (B) Bar graphs summarize the MFI±SD of CCR2, CCR4, CCR6, CCR7 and CD86 (n = 4–6). (DOCX) [file ppat.1006462.s011.docx]

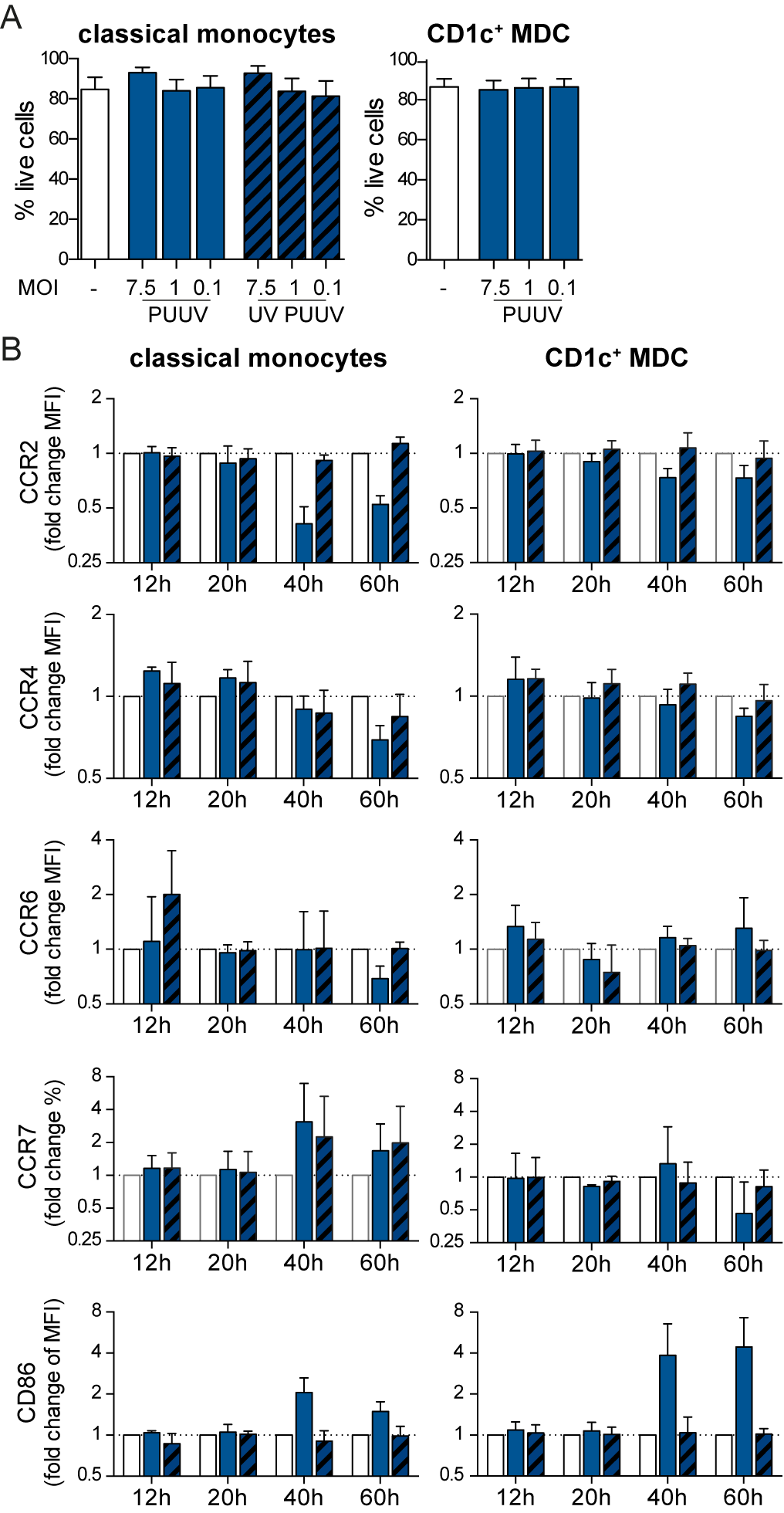


**Figure S6. Dynamic changes in expression of chemokine receptors on classical monocytes and CD1c^+^ MDCs upon exposure to PUUV *in vitro*.** (**A**) Human CM and CD1c^+^ MDCs were isolated from peripheral blood of healthy volunteers. Cells were left unexposed (white), exposed to PUUV (ocean blue) or UV PUUV (patterned ocean blue) for 2 hours at an MOI of 7.5, 1 or 0.1. Excess viruses were removed and cells were subsequently infected for 12-60 hours. Viability of cells at 24 hours was assessed by flow cytometry based on a LIVE/DEAD dye. Graphs show mean±SD viability of CM (left panel, n=3) or CD1c^+^ MDC (right panel, n=3). (**B**) Bar graphs summarize the MFI±SD of CCR2, CCR4, CCR6, CCR7 and CD86 (n=4-6).
